# Impairment of adrenergically-regulated thermogenesis in brown fat of obesity-resistant mice is compensated by non-shivering thermogenesis in skeletal muscle

**DOI:** 10.1016/j.molmet.2023.101683

**Published:** 2023-01-30

**Authors:** Petra Janovska, Petr Zouhar, Kristina Bardova, Jakub Otahal, Marek Vrbacky, Tomas Mracek, Katerina Adamcova, Lucie Lenkova, Jiri Funda, Tomas Cajka, Zdenek Drahota, Sara Stanic, Arild C. Rustan, Olga Horakova, Josef Houstek, Martin Rossmeisl, Jan Kopecky

**Affiliations:** 1 Laboratory of Adipose Tissue Biology, Institute of Physiology of the Czech Academy of Sciences, Videnska 1083, 142 00, Prague, Czech Republic; 2 Laboratory of Developmental Epileptology, Institute of Physiology of the Czech Academy of Sciences, Videnska 1083, 142 20, Prague, Czech Republic; 3 Laboratory of Bioenergetics, Institute of Physiology of the Czech Academy of Sciences, Videnska 1083, 142 20, Prague, Czech Republic; 4 Laboratory of Translational Metabolism and Laboratory of Bioactive Lipids, Institute of Physiology of the Czech Academy of Sciences, Videnska 1083, 142 20, Prague, Czech Republic; 5 Department of Physiology, Faculty of Science, Charles University in Prague, Vinicna 7, 128 44, Prague, Czech Republic; 6 Section for Pharmacology and Pharmaceutical Biosciences, Department of Pharmacy, University of Oslo, Sem Sælands vei 3, 0371, Oslo, Norway

**Keywords:** Non-shivering thermogenesis, Sarcolipin, Mitochondrial supercomplex, Skeletal muscle, Brown adipose tissue, Obesity, AJ, A/J01aHsd murine strain, AMPK, adenosine monophosphate-activated kinase, B6, C57BL/6 J murine strain, BAT (iBAT), (interscapular) brown adipose tissue, CA, cold-acclimation or cold-acclimated, CL, CL316.243 (β_3_-adrenergic agonist), COX, cytochrome *c* oxidase, DNL, *de novo* lipogenesis, EMG, electromyography, FAox, fatty acid oxidation, FA, fatty acids, FDG, 18F-fluorofeoxyglucose, INCA, indirect calorimetry, MMG, mechanomyography, MS-LFQ, mass spectrometry – label-free quantification, NE, norepinephrine, NST, non-shivering thermogenesis, PCA, principal component analysis, PET/CT, positron emission tomography / computed tomography, PRCF, percent relative cumulative frequency, qPCR, quantitative PCR, RQ, respiratory quotient, SERCA, sarco(endo)plasmic reticulum calcium ATPase, SNS, sympathetic nervous system, TAG, triacylglycerols, T_b_, core body temperature, UCP1, uncoupling protein 1, WA, warm-acclimation or warm-acclimated, WAT (eWAT, iWAT, rpWAT), (epididymal, inguinal, retroperitoneal), white adipose tissue

## Abstract

**Objective:**

Non-shivering thermogenesis (**NST**) mediated by uncoupling protein 1 (**UCP1**) in brown adipose tissue (**BAT**) can be activated *via* the adrenergic system in response to cold or diet, contributing to both thermal and energy homeostasis. Other mechanisms, including metabolism of skeletal muscle, may also be involved in NST. However, relative contribution of these energy dissipating pathways and their adaptability remain a matter of long-standing controversy.

**Methods:**

We used warm-acclimated (30 °C) mice to characterize the effect of an up to 7-day cold acclimation (6 °C; CA) on thermoregulatory thermogenesis, comparing inbred mice with a genetic background conferring resistance (A/J) or susceptibility (C57BL/6 J) to obesity.

**Results:**

Both warm-acclimated C57BL/6 J and A/J mice exhibited similar cold endurance, assessed as a capability to maintain core body temperature during acute exposure to cold, which improved in response to CA, resulting in comparable cold endurance and similar induction of UCP1 protein in BAT of mice of both genotypes. Despite this, adrenergic NST in BAT was induced only in C57BL/6 J, not in A/J mice subjected to CA. Cold tolerance phenotype of A/J mice subjected to CA was not based on increased shivering, improved insulation, or changes in physical activity. On the contrary, lipidomic, proteomic and gene expression analyses along with palmitoyl carnitine oxidation and cytochrome *c* oxidase activity revealed induction of lipid oxidation exclusively in skeletal muscle of A/J mice subjected to CA. These changes appear to be related to skeletal muscle NST, mediated by sarcolipin-induced uncoupling of sarco(endo)plasmic reticulum calcium ATPase pump activity and accentuated by changes in mitochondrial respiratory chain supercomplexes assembly.

**Conclusions:**

Our results suggest that NST in skeletal muscle could be adaptively augmented in the face of insufficient adrenergic NST in BAT, depending on the genetic background of the mice. It may provide both protection from cold and resistance to obesity, more effectively than BAT.

## Highlights

• Cold acclimation led to cold resistance and similar UCP1 level in B6 and A/J mice.
• Adrenergic non-shivering thermogenesis (NST) in BAT was induced only in B6 mice.
• Shivering did not compensate for the lack of induction of BAT NST in A/J mice.
• Adaptive NST in skeletal muscle replaced defective BAT activation in A/J mice.
• Muscle NST could contribute to obesity resistance of A/J mice.

## Graphical abstract

**Figure:**
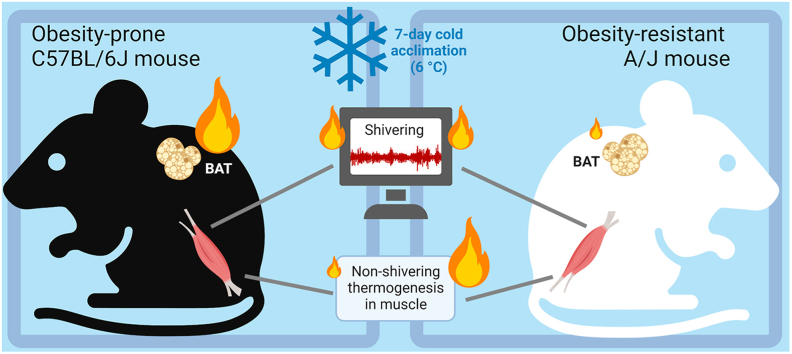

## 1 Introduction

Energy homeostasis reflects a balance between energy intake and energy expenditure. In birds and mammals, control of energy expenditure is important for maintaining a stable body temperature, and in all organisms for regulation of body weight [1]. Energy expenditure also depends on “metabolic efficiency”, which is a target for the treatment of both obesity and cachexia [2]. Still, the mechanisms involved in the regulation of energy expenditure require better characterization.

When birds and mammals are exposed to cold, besides activation of various heat-saving mechanisms [[1], [2], [3], [4], [5], [6], [7]], they enhance energy expenditure by shivering [8]. However, shivering compromises physical activity [9,10]. Long-term exposure (i.e. acclimation) to cold environment results in adaptive increase in the capacity for non-shivering thermogenesis (**NST**) and, therefore, the cessation of shivering [1,3,4,8,11]. Identical pathways of energy expenditure might be involved in both cold- and diet-induced NST [[12], [13], [14]].

A unique form of adaptive and facultative NST [14] developed only in mammals, which depends on mitochondrial uncoupling protein 1 (**UCP1**) in brown adipose tissue (**BAT**) [1]. UCP1 is also present in inducible adipocytes interspersed in white adipose tissue (**WAT**) depots [15], called brite/beige adipocytes [16,17]. Induction of UCP1-mediated thermogenesis using β_3_-adrenergic agonist [18] or transgenesis [19,20] could reduce obesity in animals. The former approach worked even in humans, but cardiovascular side effects prevented its clinical use [21]. Therefore, UCP1-independent mechanisms of NST and their potential to counteract obesity and improve metabolic health [2,[5], [6], [7],^22^,^23^] need to be explored.

NST in skeletal muscles is evolutionary older than that in BAT. It represents the main NST mechanism in birds [1,3,4,9] and it probably also operates in mammals, including humans [8]. Its capacity may be substantial, since the muscle can account for 20–30% of the total oxygen uptake in the resting state [1,3], which could affect pathogenesis of obesity [24]. Several mechanisms are likely to contribute to muscle NST, namely (i) UCP1-independent mitochondrial proton leak [8,11]; (ii) impaired thermodynamic efficiency of Na^+^/K^+^-ATPase in the plasma membrane [25,26] or components of mitochondrial respiratory chain [27,28]; (iii) muscle tonus [29,30]; (iv) futile substrate cycling between *de novo* lipogenesis (**DNL**) and fatty acid (**FA**) oxidation (**DNL/FAox** cycle) controlled by leptin – AMP-activated protein kinase (**AMPK**) axis [[31], [32], [33], [34]]; and (v) uncoupling of sarco (endo)plasmic reticulum calcium ATPase (**SERCA**) pump activity by sarcolipin [8,9,[35], [36], [37]]. Gene targeting experiments in mice indicated that both sarcolipin and UCP1 may be required for thermogenesis in cold [38,39]. Moreover, loss of sarcolipin resulted in obesity, while overexpression of its gene enhanced NST and induced resistance to diet-induced obesity [40]; reviewed in [9]. Muscle sarcolipin level is increased in mice with ablation of either interscapular BAT (**iBAT**) or UCP1 [38,41,42]. Thus, BAT and muscle may represent synergistic and potentially even partially redundant components of thermoregulatory NST [9,11]. However, while the adaptive nature of NST is widely accepted for BAT, for skeletal muscle this remains a matter of long-standing controversy [14,43,44].

Acute stimulation of NST by both diet and cold is mediated primarily by sympathetic nervous system (**SNS**), affecting mostly heat production in BAT [1,7]. This is in accordance with the notion that only UCP1-based thermogenesis is adrenergically regulated [14,43] and that UCP1 is essential for adaptive adrenergic NST [45]. Control of UCP1-independent NST is probably more complex. Mechanisms underlying its acute regulation are not known. Its long-term control depends on leptin [13,46,47], which acts both centrally and directly *via* leptin receptors in the muscle [34], thyroid hormones [48,49], insulin, FGF21 [50] and other factors (reviewed in [1,3,4,9]). Both thyroid hormones and leptin were required for thermogenesis in the absence of UCP1 [31].

Thermoregulatory thermogenesis depends on oxidation of FA, which are released from triacylglycerols (**TAG**) contained in WAT, channelled to the liver for incorporation into VLDL-TAG, and eventually serving as fuel for oxidation in extra-adipose tissues. Thus, the inter-organ communication *via* metabolic fluxes is essential [8,[51], [52], [53]]. We have shown [52] that 7-day-cold acclimation (**CA**) to 6 °C of obesity-resistant A/J01aHsd (**AJ**) and obesity-prone C57BL/6 J (**B6**) mice pre-adapted to 30 °C resulted in a stronger induction of WAT metabolism in AJ mice, including DNL, TAG/FA cycling, lipolysis and FA release into circulation. This suggested higher induction of thermogenesis fuelled by WAT-released FA in extra-adipose tissue(s) in AJ mice [52]. Previous studies suggested stronger activation of UCP1 gene (***Ucp1***) expression by cold, β_3_-adrenergic agonist [15] and high-fat diet [54] in AJ than in B6 mice. However, muscle NST was also activated by weaning to high-fat diet in AJ but not B6 mice [33]. Therefore, the thermogenic mechanism(s) behind the strain-specific activation of WAT metabolism by CA remain to be identified. These mechanisms could also affect propensity to obesity, since AJ mice are resistant and B6 mice are prone to obesity [15,18,33,55].

Here, warm-acclimated mice of both AJ and B6 strains were subjected or not to CA as before [52] while the thermogenic mechanisms were characterized. Our results reveal surprising impairment of adaptive adrenergic NST in BAT of AJ mice. They suggest its compensation by adaptive NST in skeletal muscle, which could contribute to both cold tolerance and obesity-resistance of AJ mice.

## 2 Materials and methods

### 2.1 Ethics statement

Animal experiments were approved by the Institutional Animal Care and Use Committee and the Committee for Animal Protection of the Czech Academy of Sciences (Approval Number: 81/2016, 48/2019, and 43-2022-P).

### 2.2 Mouse models

Experiments were conducted using AJ (Harlan Laboratories UK Ltd.) and B6 (Taconic Biosciences, Denmark) male mice, similarly as before [52]. After their arrival at 6 weeks of age, mice were caged in groups of 3 and kept for 2 weeks in a controlled environment, i.e. at 22 °C, 50% humidity, and 12 h/12 h light/dark cycle (light from 6 a.m.), with drinking water and diet ad libitum. Mice were fed standard chow (extruded Ssniff R/M−H from Ssniff Spezialdiaten GmbH, Soest, Germany; metabolizable energy 13 MJ/kg). Thereafter, mice were maintained close to thermoneutrality (at 30 °C) for 2 weeks, and for 7 more days either at 30 °C (**WA**) or subjected to cold in groups of 3–4 (6 °C; CA), before *in vivo* phenotyping using indirect calorimetry or positron emission tomography (**PET**) imaging (see below). Alternatively, mice were killed under ether anesthesia (between 8 a.m. and 10 a.m.) and iBAT, inguinal WAT (**iWAT**; see [56]), retroperitoneal WAT (**rpWAT**), epididymal WAT (**eWAT**) and skeletal muscle (*musculus gastrocnemius*) were dissected, and flash-frozen in liquid N_2_ and stored at −80 °C for further analyses.

### 2.3 Cold endurance and adrenergically–stimulated oxygen consumption

These measurements were performed using an 8-chamber indirect calorimetry system (**INCA**; Somedic, Horby, Sweden) described before [33]. Oxygen consumption (ml O_2_/min) and carbon dioxide production (ml CO_2_/min) were recorded every 2 min under a constant airflow (1000 ml/min) in each chamber simultaneously. The level of substrate partitioning was estimated by calculating respiratory quotient (**RQ**), i.e. the ratio between produced CO_2_ and consumed O_2_. To compare subtle differences between subgroups, the percent relative cumulative frequency (**PRCF**) curves were also drawn, based on RQ values pooled from all animals within a given subgroup [10,33,57]; the curves were fitted with sigmoidal variable slope (four parameters) function using GraphPad Prism v. 8.4.2. The INCA system was extended for telemetry of core body temperature (**T**_**b**_) and physical activity of the animals. For these measurements, some mice were intraperitoneally implanted with Mini-Mitter transponders (Respironics, PA, USA) at 5 weeks of age, i.e. before the WA/CA.

For cold endurance (tolerance or sensitivity) assessment, a partially modified protocol published by Meyer et al. [58] was used. At 10 a.m., awake WA and CA mice of both strains, maintained at the appropriate temperature (30 °C and at 6 °C, respectively) were placed singly into the INCA chambers without food, with the temperature controlled at 5 °C. The INCA parameters, T_b_ and physical activity were recorded for 4 h or until the T_b_ dropped below 28 °C (in this case, the animals were withdrawn from cold immediately). Baseline values of the parameters above were evaluated at 33 °C during 60 min of the measurements using different groups of mice.

To evaluate adrenergically-stimulated metabolic rate, mice were anesthetized using intraperitoneal injection of pentobarbital (80 and 100 μg pentobarbital/g body weight for B6 and AJ mice, respectively), and placed into the INCA chambers, with the temperature controlled at 33 °C. Baseline values of oxygen consumption (as well as core T_b_ when indicated) were recorded for at least 20 min before the animals were injected s. c. with either β_3_-adrenergic agonist CL316,243 (**CL;** 1 μg/g body weight) or mixed adrenergic agonist norepinephrine (**NE**; bitartrate monohydrate; 1 μg/g body weight) [45] and measurement continued for 80 min after the injection.

### 2.4 Body insulation and thermal conductance

Single-caged AJ and B6 mice implanted with Mini-Mitter transponders (see 2.3) were acclimated to 30 °C for 2 weeks. Subsequently, the mice of both genotypes were randomly assigned into 3 experimental groups and kept for following 2 weeks at 30 °C, 22 °C, or 8 °C in measuring chambers of indirect calorimetry system Phenomaster (TSE, Germany).

Thermal conductance was assessed in all the above mentioned experimental groups after 2-week acclimation to the respective temperature. Mean consumption of O_2_, production of CO_2_, and T_b_ data were obtained during 12-h light phase. Energy expenditure (kcal/h) was calculated according to formula: Energy expenditure (kcal/h) = 3.9 x O_2_ consumption (l/h) + 1.1 x CO_2_ production (l/h) as before [59]. Thermal conductance was calculated according to formula: Thermal conductance [kcal/h/°C] = EE [kcal/h]/(T_b_ [°C] – ambient temperature [°C]) [60].

To assess body insulation, infrared photos of body surface of the AJ and B6 mice kept at 8 °C were taken by thermal imager Testo890 (Testo, Germany). This procedure was performed between 11 and 12 a.m., three times during the experiment: before start of acclimation to 8 °C (i.e. at 30 °C), and then 2 and 7 days after the start of 8 °C acclimation. Hair surface temperature in upper back area was quantified using IRSoft software as the hottest point in the region. Photos with skin surface directly exposed (due to disturbances of fur caused by handling) were excluded from evaluation.

### 2.5 RNA isolation and gene expression

Total RNA was isolated from various fat depots and gastrocnemius muscle, and transcript levels were assessed using quantitative real-time PCR (**qPCR**) as described [52]. Data were normalized using geometric mean of several housekeeping genes (in adipose tissues: *Eef2, beta actin*, and *Rn18s*; in muscle: *Hprt* and *Rn18s*). See S4 Table for PCR primer sequences and gene names**.**

### 2.6 Quantification of UCP1 using immunoblots

UCP1 protein was quantified in homogenates of iBAT, rpWAT and iWAT using Western blotting as described before [61,62], mouse anti-human UCP1 (MAB6158; R&D system) and infrared dye-labelled secondary antibodies (the Odyssey IR Imaging Systems; Li-Cor Biosciences, Lincoln, NE, USA). Signals on different blots were compared using a standard of iBAT homogenate prepared from 2-month-old B6-CA mice. The signal of glyceraldehyde-3-phosphate dehydrogenase detected using specific antibodies (1:1000, Cell Signalling, MA, USA) was used as the control of the loading. Results are expressed in arbitrary units (A.U.) per mg of tissue and also recalculated to whole depot.

### 2.7 Native electrophoresis

Tissue homogenates (10%, w/v) were prepared at 4 °C from the frozen gastrocnemius muscle in KCl based medium (150 mM KCl, 50 mM Tris-HCl, 2 mM EDTA ∼ 7.4 pH) containing protease inhibitor cocktail, (PIC 1:1000, Sigma P8340) using glass-teflon homogenizer and filtered through fine mesh [63]. Tissue homogenates were centrifuged 15 min at 20 000 *g* at 4 °C to obtain membrane fraction. These pellets were resuspended in buffer containing 50 mM NaCl, 2 mM 6-aminohexanoic acid, 50 mM imidazole, 1 mM EDTA, protease inhibitor cocktail, PIC (1:1000) pH 7, solubilized with digitonin (4 g/g protein) for 20 min on ice and centrifuged for 20 min at 30 000 *g*. To the resulting supernatants, 5% glycerol and Coomassie Brilliant Blue G-250 (dye/detergent ratio 1:8) were added and samples were subsequently analyzed by Blue-Native electrophoresis using 4–13% polyacrylamide gradient mini gels and the imidazole buffer system [64]. Gels were blotted onto PVDF (polyvinylidene difluoride) membrane (Immobilon FL 0.45 μm, Merck) by semi-dry electro-transfer (1 h at 0.8 mA/cm2) using a Transblot SD apparatus (Bio-Rad). PVDF membranes were washed for 5 min in TBS (150 mM Tris- HCl, 10 mM NaCl; pH 7.5) and blocked in 5% (w/v) fat-free dry milk diluted in TBS for 1 h. Then, the membranes were washed 3 × 10 min in TBST (TBS with 0.1% (v/v) detergent Tween-20). For Western blot immunodetection, the membranes were incubated in primary antibody (2 h at room temperature or overnight at 4 °C). We used antibodies to subunits of respiratory Complex I (NDUFB8, Abcam ab110242), Complex II (SDHA, Abcam ab14715), and Complex III (Core 2, ProteinTech 14742-1-AP). For quantitative detection, the corresponding infra-red fluorescent secondary antibodies (Alexa Fluor 680, Thermo Fisher Scientific; IRDye 800, LI-COR Biosciences) were used. Detection was performed using the fluorescence scanner Odyssey (LI-COR Biosciences) and signals were quantified by ImageLab 6.0 software (Bio-Rad). Results were normalised to the SDHA signal and expressed as relative difference against AJ-WA group.

### 2.8 Glucose uptake *in situ*

Positron emission tomography - computed tomography (**PET/CT**) imaging was used to assess *in situ* adrenergically – stimulated glucose uptake in iBAT similarly as before [65]. Both WA and CA mice were fasted for 4–5 h at the respective ambient temperature before the measurement at 22 °C. Mice were injected s. c. by CL (1 μg/g body weight) and 40 min after the injection, mice were anesthetized using i. p. injection of pentobarbital (80 and 100 μg pentobarbital/g body weight for B6 and AJ mice, respectively), placed on a heating pad to prevent hypothermia, and injected by ^18^F-fluorodeoxyglucose (**FDG;** 10 MBq; diluted in saline to a final volume 0.2 ml), applied to a lateral tail vein. Blood glucose levels did not differ among the experimental groups at the time of FDG injection (not shown). After 60 min, PET imaging was performed using μPET/CT Albira (Bruker Corp., Belgium; see below). All the animals were subjected to a CT (45 kV 0.4 A) scan after a static PET scan for anatomical delineation of PET image. PET images were reconstituted using Albira Reconstructor software and evaluation of FDG uptake was performed in PMOD v. 3.403 (PMOD Technologies, Ltd., Switzerland). BAT volume was determined by thresholding CT image and the resulting mask was applied on PET data. The resulting quantitative data were expressed as SUV [%ID]. The imaging procedure lasted for 45 min.

### 2.9 Proteomics

Samples for mass spectrometry label-free quantification (**MS-LFQ**) were prepared as described before [66]. Briefly, samples of iBAT were solubilized using sodium deoxycholate (1% (w/v) final conc.), reduced with TCEP [tris(2-carboxyethyl)phosphine], alkylated with MMTS (S-methyl methanethiosulfonate), digested sequentially with Lys-C and trypsin and extracted with ethylacetate saturated with water. Thereafter the samples were desalted on Empore C18 columns, dried in Speedvac and dissolved in 0.1% TFA +2% acetonitrile. About 1 μg of peptide digests were separated on 50 cm C18 column using 2.5 h elution gradient and analyzed in a DDA mode on a Orbitrap Fusion Tribrid (Thermo Scientific) mass spectrometer. Resulting raw files were processed in MaxQuant (v. 1.5.3.28) with LFQ algorithm MaxLFQ.

### 2.10 Evaluation of muscular thermogenic activity (“shivering”)

Mice pre-acclimated at 30 ^ο^C were anesthetized using isoflurane, and bipolar electrodes in the form of a loop fabricated from 50 μm diameter teflon-insulated silver wire (AM Sytems; 787 000) were placed upon the surface of nuchal muscles and a grounding electrode on the left side of the head. Leads were soldered into the small connector (Mill Max, USA) and fixed with dental acrylic to the skull together with two anchor screws. Five days after the surgery, the electromyography (**EMG**) and mechanomyography (**MMG**) signals were recorded in mice at thermoneutrality for 30 min (between 9:00 and 11:00). Subsequently, the mice were transferred to 6 °C and signals were recorded and compared after acute cold. After 2 days and 7 days of cold exposure, signal was recorded in the mice again for 60 min (approximately at 12–16:00). Animals were during the measurement connected to a custom-made miniature headstage (adapted version of electroencephalography headstage used in epilepsy research on mice) containing high sensitive three-axis accelerometer (ADXL335, Analog Devices, USA). The signal from both accelerometer and EMG was preprocessed (gain 5000x, highpass and antialiasing filter 10–3000 Hz) and digitized at 20 kHz with 16bit resolution directly on the headstage microcircuit to maximize signal to noise ratio. Digital signal was transferred into the PC *via* USB with ultra-thin high flexible cable with attached 12-channel commutator (MOOG, Germany) and stored on the hard-drive for off-line analysis. Data were analyzed in Spike2 software (CED, UK) and Matlab software (Mathworks, Nattick, USA). Amplitude (rectified and smoothed electromyograph and acceleration, root mean square – RMS) analysis allowed for evaluation of muscle activity.

“Mean muscular activity” calculated as mean acceleration across the whole period excluding initial and final 2.5 min was evaluated as RMS (time constant 0.1 s). For a more detailed analysis, segments corresponding to periods with minimal or no locomotor activity lasting for 4 min were selected and the lowest RMS based on moving average for 15 s within the selected 4-min segments was identified as “low-intensity muscular activity” (as done before for electromyography in [43]).

### 2.11 Palmitoyl carnitine oxidation and COX activities in gastrocnemius muscle

10% muscle homogenate (w/v) was prepared from frozen tissue samples using teflon – glass homogenizer and the ice-cold K-medium (80 mM KCl, 10 mM Tris–HCl, 5 mM K_2_HPO_4_, 1 mM EDTA, 3 mM MgCl_2_, pH 7.2). The activities were followed as the rate of oxygen consumption using Oroboros Oxygraph (Oroboros, Austria) and the homogenate aliquots (see below) incubated in 2 ml of the same K-medium at 30 °C. Both activities were expressed as pmol oxygen/sec/mg protein.

Palmitoyl carnitine oxidation was determined [67] using 2.5 mg homogenate protein, in the presence of 10 μM cytochrome *c*, 2.5 mM malate and 1.5 mM ADP. Finally, palmitoyl carnitine was added as a respiratory substrate in two steps to reach the final concentration of 3.1 μM and 10 μM (the first dose of palmitoyl carnitine was fully oxidized within minutes, the second dose resulted in stable oxygen consumption at a similar level to the lower dose). Finally, the quality of mitochondria in the homogenate was tested by adding 10 mM succinate (see S8 Fig for the representative curve).

Cytochrome *c* oxidase (**COX**) activity was recorded as previously [68] using 0.3 mg of homogenate protein. Subsequently, the following compounds were added (final concentration): (i) cytochrome *c* (2 mM), (ii) Na-ascorbate (5 mM) and N,N,N′,N′-tetramethyl-p-phenylenediamine dihydrochloride (TMPD; 0.5 mM); and (iii) KCN (0.25 mM). KCN-insensitive oxygen uptake was subtracted from the oxygen consumption rate after addition of TMPD.

### 2.12 Protein concentration

Protein content was determined by bicinchoninic acid [69].

### 2.13 Metabolomics and lipidomics

Metabolomic and lipidomic analyses were performed on gastrocnemius muscle extracts (see Supplementary Methods).

### 2.14 Statistical analysis

All data in figures are expressed as means ± SEM. In column graphs, individual values are plotted as well. For the statistical tests, see the legend to each figure. To find fundamental relations between data sets, principal component analysis (**PCA**) was performed using MetaboAnalyst 4.0 and 5.0 web portal [70].

## 3 Results

### 3.1 Comparable cold endurance of B6 and AJ warm-acclimated mice

To compare the thermoregulatory mechanisms engaged in cold defence of B6 and AJ mice, animals of both genotypes were housed at thermoneutrality (at 30 °C) for 2 weeks and maintained for 7 additional days either at 30 °C (WA) or 6 °C (CA). This relatively short-term acclimation protocol resulted previously in almost complete change in the cold adaptive iBAT proteome in B6 mice [71]. B6 as compared to AJ mice were heavier (S1 Table) while plasma levels of both TAG and non-esterified FA were higher in AJ mice, independent of CA [52].

**Figure 1:**
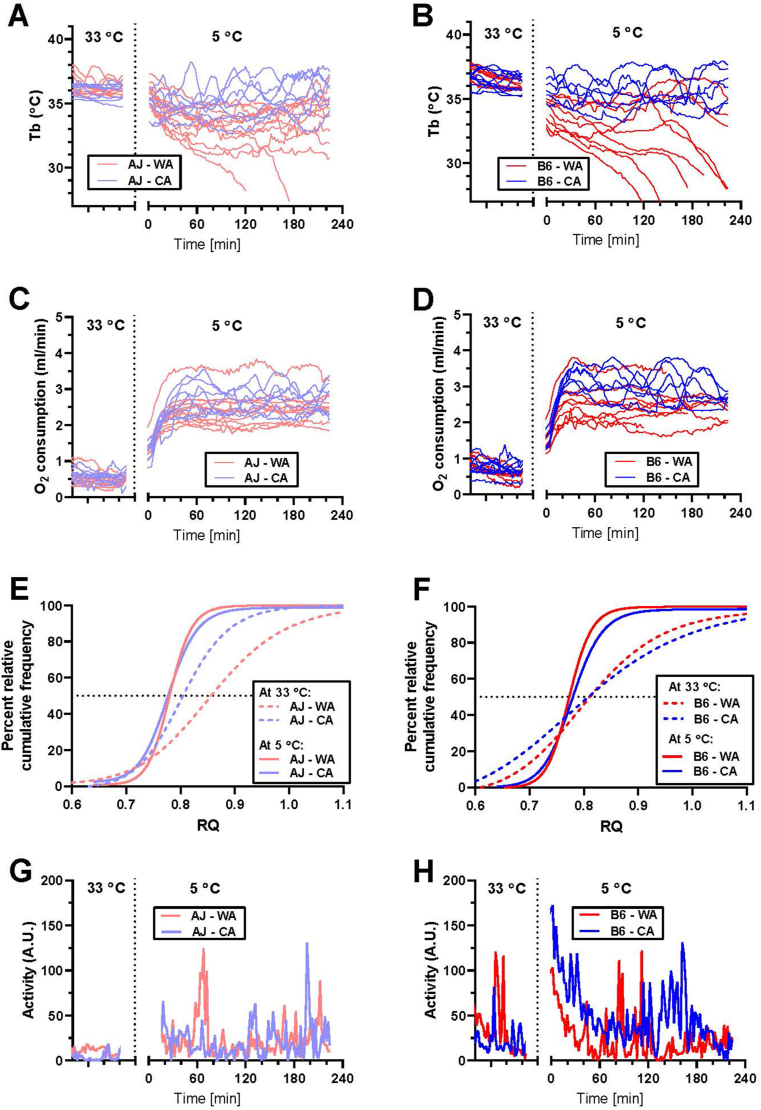
**Lower cold endurance of B6 compared to AJ warm-acclimated mice and rescue of the endurance by CA**. Whole-body measurements were performed using AJ and B6 mice acclimated to a thermoneutral housing temperature (30 °C; WA) or to cold (6 °C; CA), at 33 °C and 5 °C, respectively, using different groups of mice; for *n* in various groups, see legend to S2 Table. **(A** and **B)** Core body temperature (T_b_) of AJ **(A)** and B6 mice **(B)** measured at 33 °C (left part of the graphs) or at 5 °C (right part of the graphs); data for individual mice are plotted (for mean curves, see S1A and S1B Fig). **(C** and **D)** Oxygen consumption of AJ **(C)** and B6 mice **(D)** measured at 33 °C (left part of the graphs) or at 5 °C (right part of the graphs); data for individual mice are plotted (for mean curves, see S1C and S1D Fig). **(E** and **F)** Percent relative cumulative frequency (PRCF) of respiratory quotient (RQ) of AJ **(E)** and B6 mice **(F)**; the PRCF curves pooled from RQ values from all animals in a given group. **(G** and **H)** Physical activity of AJ **(G)** and B6 **(H)** mice; mean curves for whole experimental group are plotted. See S2 Table for the mean values of the measured parameter, and the number of the animals.

Mice with abdominally implanted transponders for telemetry of T_b_ and physical activity were individually placed in INCA chambers with drinking water but without food (Figure 1A–H; S1A – S1D Fig). When measured at 33 °C, AJ mice exerted lower (∼0.3–0.4 °C) mean T_b_ compared to B6 mice, but there was no difference in T_b_ between the WA and the CA mice (S2 Table). To assess cold endurance, mice were placed in a precooled chamber and the measurements proceeded at 5 °C for 4 h or until those animals that developed severe hypothermia (T_b_ < 28 °C) were rescued from the cold (Figure 1A,B, Fig S1A and S1B). During the first hour, T_b_ decreased in all the groups, with a more pronounced decline in the WA vs. the CA mice, resulting in ∼0.7 °C lower T_b_ values in AJ mice (S2 Table). During following 3 h, several WA mice (2 out of 10 AJ, and 6 out of 9 B6) developed hypothermia (Fig 1B) suggesting higher cold sensitivity of B6 mice, but the interstrain difference was not statistically significant (p = 0.07 according to Fisher's exact test for development of hypothermia). In contrast, all the CA mice of both strains were able to maintain stable T_b_ (∼1 °C lower compared with the baseline values at 33 °C; S2 Table, Figure 1A–B, and S1A – S1B Fig).

When measured at 33 °C, mean metabolic rate of AJ mice (i.e. oxygen consumption per animal) was ∼1.3–1.4-fold lower as compared with B6 mice, independent of CA (S2 Table). During the first hour at 5 °C, mean metabolic rate of all mice increased ∼4 – 5-fold and it stayed elevated during 4 h of the measurement. In B6 but not in AJ mice, CA augmented the stimulatory effect of the acute cold exposure on O_2_ consumption (Figure 1C,D; S2 Table).

Mean levels of RQ, the marker of substrate partitioning, were similar across all the groups within the two measuring temperatures, and they were lower at 5 °C vs. 33 °C indicating greater oxidation of lipids in the cold (S2 Table). Next, robust analysis of the RQ data was performed by drawing PRCF curves based on pooled data from all the animals within a given group [10,33,57]. A leftward shift in the PRCF of RQ toward lower RQ in response to acute cold exposure (i.e. 33 °C vs. acute 5 °C) was observed in both AJ (Fig 1E) and B6 (Fig 1F) mice. This indicated a higher frequency of lower RQ values. Besides acute cold exposure, CA also caused a leftward shift of PRCF curve but only when measured at 33 °C, and only in AJ mice (Fig 1E), suggesting a strain-specific adaptive induction of lipid oxidation. Apparently, this phenotype was masked by the strong increase in lipid oxidation in mice of both strains in response to acute cold exposure.

At 33 °C, physical activity was higher in B6 mice independent of housing temperature, and it was similar in mice of all the groups when measured at 5 °C, except for a relatively high physical activity in the B6-CA mice (Figure 1G,H; S2 Table).

Taken together, the above results indicate two qualitatively different adaptive responses to CA in the two genotypes: (i) increase in the capacity for energy expenditure in B6 mice, resulting (in part) from physical activity (Figure 1D,H); and (ii) switch in fuel partitioning from glucose to lipid oxidation in AJ mice, which was apparent at 33 °C (Fig 1E), and which was not associated with a significant increase in energy expenditure of acutely cold-exposed mice (Figure 1C, S1C Fig; see Discussion).

To learn whether release of body heat could contribute to the interstrain phenotypic differences in thermal homeostasis, body surface temperature was recorded using infrared camera at the back of the animals. These measurements were performed at 30 °C, and during consequent maintenance at 8 °C (S2 Fig A), i.e. at a temperature by 2 °C higher than used during the cold endurance test, to ensure survival of singly caged WA mice in cold. As expected, mice showed higher body surface temperature at 30 °C than in cold (S2 Fig A and B). However, there was no difference between AJ and B6 mice under any of the studied conditions (S2 Fig B). Next, thermal conductance, i.e. a parameter conversely related to body insulation was calculated using T_b_ and energy expenditure data obtained in mice adapted (for 2 weeks) and measured at ambient temperature of 8, 22, and 30 °C (S2 Fig C). The conductance rose with increased ambient temperature as expected [60], but it was similar in both mouse strains, independent of the ambient temperature (S2 Fig C). Thus, thermal conductance does not contribute to the qualitatively different adaptive response of AJ and B6 mice to cold (see also below).

### 3.2 Similar CA-induced levels of functional UCP1 in AJ and B6 mice

**Figure 2:**
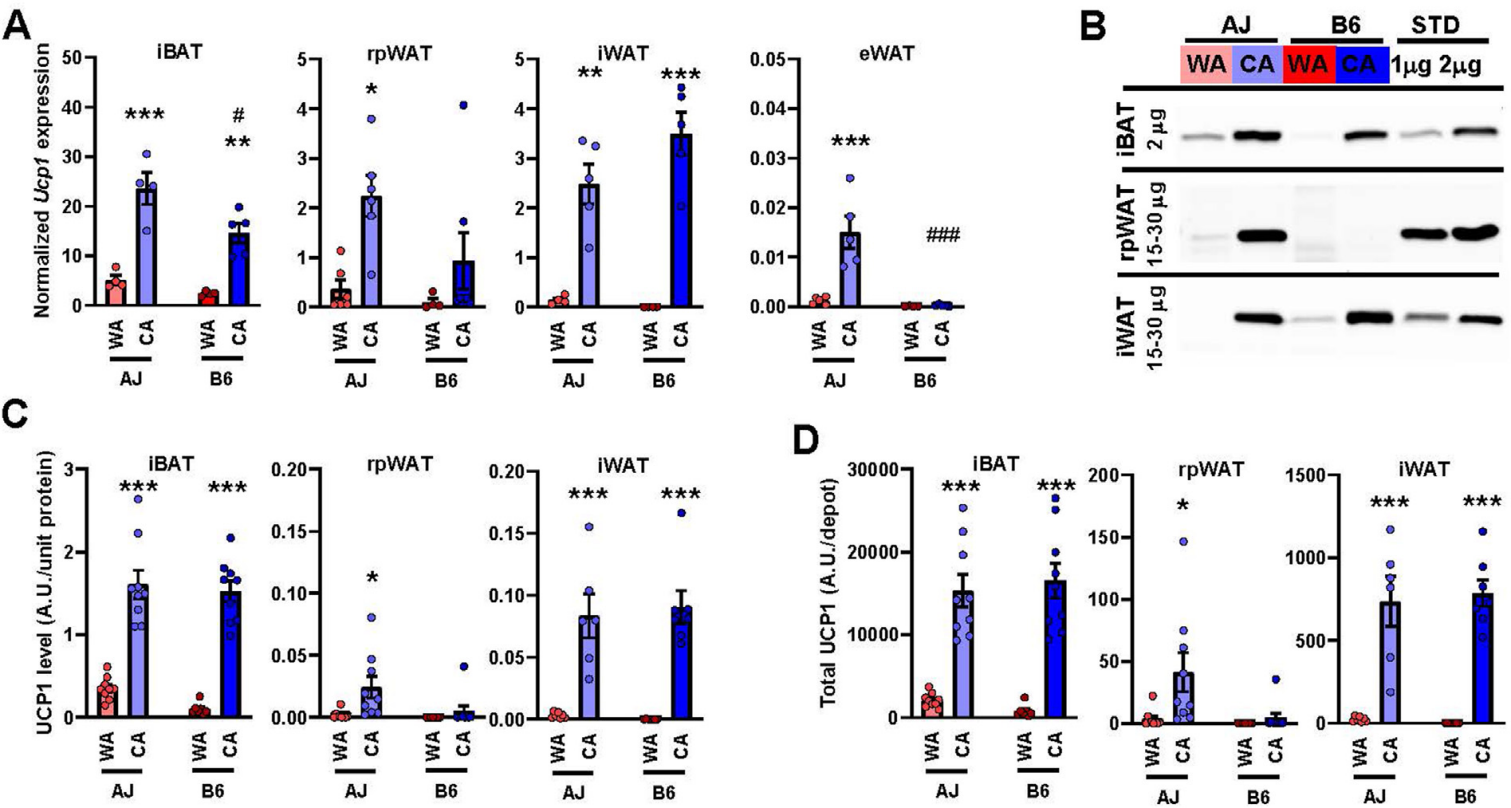
**Higher expression and content of UCP1 in fat depots of AJ than B6 warm-acclimated mice and higher induction by CA in B6 mice**. Analyses were performed using AJ and B6 mice acclimated to a thermoneutral temperature (30 °C; WA) or to cold (6 °C; CA). **(A)** Quantification of *Ucp1* transcripts in various fat depots using qPCR (data were normalized to a geometric mean of 3 housekeeping genes, see Materials and Methods); *n* = 4–7. **(B**–**D)** Quantification of UCP1 using Western blotting; *n* = 7–9; **(B)** representative blots (protein quantity of tissue homogenate analyzed is indicated); **(C)** specific content of UCP1 adjusted to protein of tissue homogenate); **(D)** total UCP1 protein per adipose tissue depot (calculated using the protein content of each fat depot; see S1 Table). iBAT, interscapular BAT; rpWAT, retroperitoneal WAT; iWAT, subcutaneous WAT from inquinal region; eWAT, epididymal WAT. Data are means ± SEM; ∗*p* < 0.05 and ∗∗∗*p* < 0.001 vs. the respective WA group; #*p* < 0.05 and ###*p* < 0.05 vs. the AJ mice (two-way ANOVA and Tukey's test). **(A, C, D)** Values for each data point can be found in S1 Data.

Next, the role of UCP1 in the adaptive increase in the thermogenic capacity by CA was studied. Using qPCR, the levels of *Ucp1* transcript were evaluated in selected fat depots of the WA mice of both genotypes with the following order of abundance: iBAT >> rpWAT = iWAT >> eWAT, and tended to be higher in AJ mice (Figure 2A, S1 Data). Levels of *Ucp1* transcript increased several fold in response to CA. The fold change was particularly large in those fat depots that exhibited low levels of *Ucp1* expression in the WA mice. Thus, the induction in iBAT was less striking, especially in AJ mice (∼5.9- vs ∼10.2-fold increase in AJ and B6 mice, respectively), resulting in higher *Ucp1* levels in AJ mice (Figure 2A). However, *Ucp1* expression in iBAT of the CA mice was still one-order of magnitude higher compared with rpWAT or iWAT, while it was negligible in eWAT.

Evaluation of UCP1 levels in iBAT, rpWAT and iWAT homogenates using Western blotting (Fig 2B) revealed a similar pattern of expression as at the transcript level. In the WA mice, the highest UCP1 levels (adjusted to homogenate protein) were found in iBAT of AJ mice (Fig 2C). The UCP1 abundance increased in response to CA in all the fat depots, reaching one- to two-order of magnitude higher levels in iBAT as compared with iWAT and rpWAT, respectively (in rpWAT, UCP1 was detected only in AJ mice; Fig 2C). The CA-induced increase of UCP1 in iBAT was less pronounced in AJ vs. B6 mice (∼2.8- vs ∼3.7-fold increase, respectively). Based on the fat depot protein content (S2 Table), total UCP1 content per depot was calculated (Figure 2D, S1 Data). Within the WA mice, the total UCP1 content in the studied depots was negligible, except for iBAT, in which it was ∼2.6-fold higher in AJ vs. B6 mice. Although the CA-induced increase in iBAT was lower in AJ than in B6 mice (∼5.2- vs. ∼7.8-fold, respectively; Fig 2D), the total UCP1 content in iBAT was similar in the CA mice of both strains, ∼20-fold higher as compared with iWAT (Fig 2D). In rpWAT, detectable levels of UCP1 were found only in the AJ-CA mice (Fig 2D).

Characterization of UCP1 function in isolated iBAT mitochondria using respirometry [72] did not reveal any substantial difference between the AJ-CA and the B6-CA mice (S3A and S3B Fig). Thus, both the induction of UCP1-mediated oxygen consumption in the presence of pyruvate and malate by CA, documenting thermogenic activity of UCP1, and its inhibition by GDP, were similar in mice of both genotypes.

Taken together, the above results unexpectedly showed similar levels of functional UCP1 in CA-induced iBAT, by far the most important site of UCP1-mediated thermogenesis, in mice of both genotypes. Also, in iWAT, UCP1 content was increased to similar levels in mice of both genotypes (albeit much lower overall compared to iBAT), reflecting the likely induction of brite/beige adipocytes [[15], [16], [17]].

### 3.3 Impaired induction of adrenergically-regulated thermogenesis by CA in AJ mice

**Figure 3:**
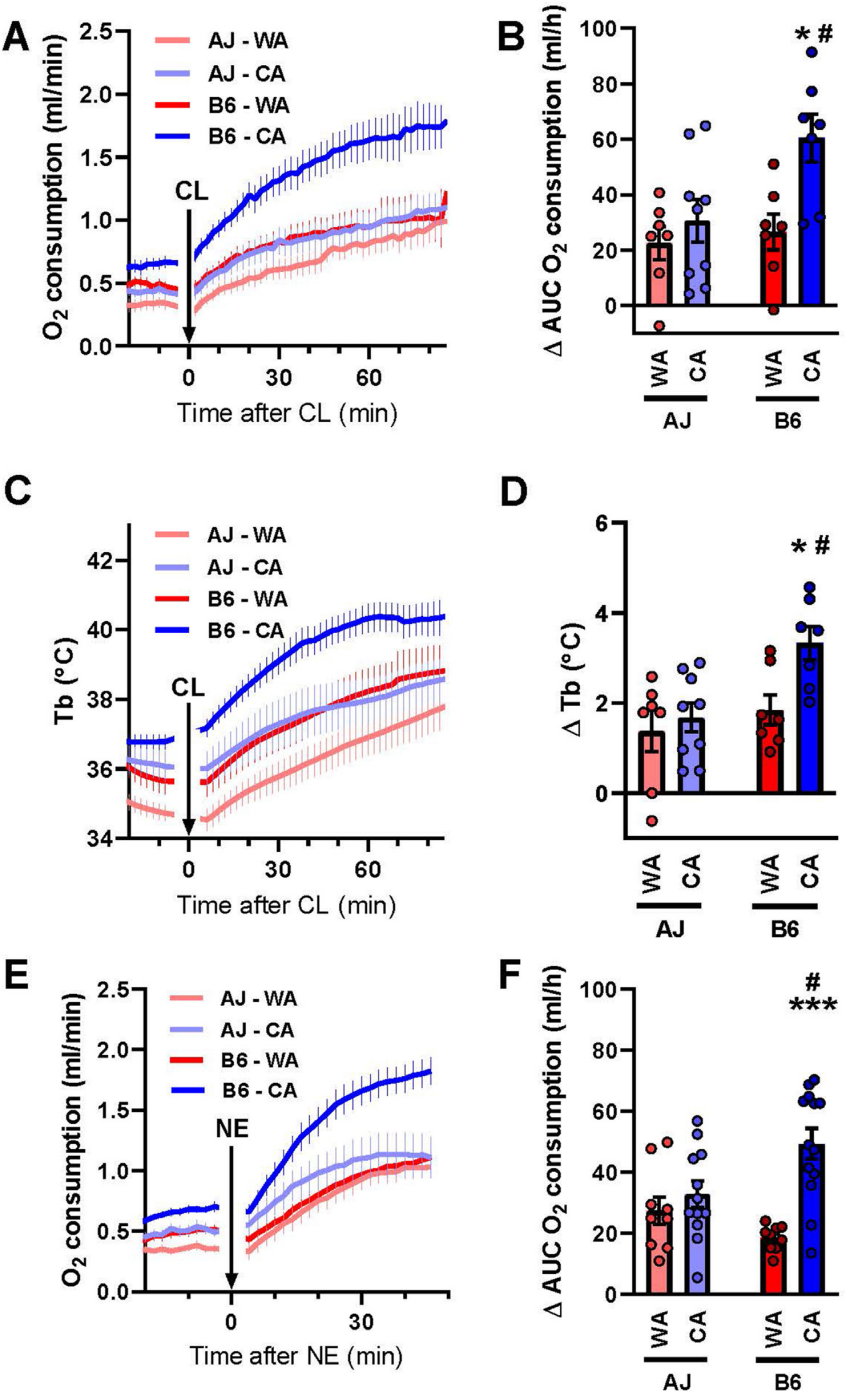
**Lower adrenergic stimulation of metabolism in AJ compared to B6 mice**. Thermoregulatory parameters of AJ and B6 mice acclimated to a thermoneutral temperature (30 °C; WA) or to cold (6 °C; CA) and measured in pentobarbital-anesthetized mice at 33 °C. **(A)** Oxygen consumption before and after injection of β_3_-adrenergic agonist CL316,243 (CL). **(B)** CL-stimulated oxygen consumption (corresponds to panel A); area under curve (AUC), of oxygen consumption during 60 min after CL injection with basal oxygen consumption (i.e. before the CL injection) subtracted (ΔAUC). **(C)** Core body temperature (T_b_) before and after injection of CL (the same animals as in panel A). **(D)** CL-stimulated T_b_ (corresponds to panel C); mean T_b_ during 30–75 min after CL injection, with the average T_b_ during 30 min before the CL injection subtracted (ΔT_b_). **(E)** Oxygen consumption before and after injection of norepinephrine (NE). **(F)** NE-stimulated oxygen consumption (corresponds to panel E; calculated as in B). Separate groups of mice were used for (i) A-D, and (ii) E and F, respectively (*n* = 7–13). **(A, C, E)** Data are means ± SEM. **(B, D, F)**. Columns and error bars represent means ± SEM; ∗*p* < 0.05 and ∗∗∗*p* < 0.001 vs. the respective WA group; #*p* < 0.05 vs. the AJ mice (two-way ANOVA and Tukey's multiple comparison test). **(B, D, F)** Values for each data point can be found in S1 Data.

Adaptive increase in capacity of NST elicited by CA, as well as stimulation of NST in response to acute cold exposure is mediated largely by the adrenergic system [1]. In turn, 60–80% of adrenergic stimulation of thermogenesis in mice depends on UCP1 [7]. Therefore, to characterize and further compare the role of UCP1 in the thermoregulatory thermogenesis in both mouse strains, we tested the acute metabolic effects of β-adrenergic receptor agonists injected s. c. into pentobarbital-anesthetized mice at 33 °C. Before injection, the metabolic rate (Figure 3A,E) and T_b_ (Fig 3C) was higher in the CA than the WA mice (*p* < 0.05; 2-way ANOVA). Injection of β_3_-adrenergic receptor agonist CL increased the metabolic rate to a similar extent in the AJ–WA, the AJ–CA and the B6–WA mice, while the stimulation in the B6–CA mice was much higher, resulting in a ∼2.4-fold higher rate 60 min after the injection vs. baseline (Figure 3A,B). A similar response was observed at the level of T_b_ (Figure 3C,D). Analogously, injection of NE that activates several subtypes of adrenergic receptors, but could be even more selective with respect to the activation of UCP1-mediated thermogenesis than CL [7], elicited a similar pattern of stimulation of metabolic rate compared to CL (Figure 3E,F).

Taken together, these data revealed a reduced response of adrenergic NST to CA in AJ mice, which contrasted with the adaptive increase in the capacity for this type of thermogenesis in B6 mice. This cannot be explained by the content of mitochondrial UCP1 (Fig 2) or its biochemical activity in mitochondria (S3 Fig).

### 3.4 Higher induction of iBAT glucose uptake in B6 mice in response to CA

**Figure 4:**
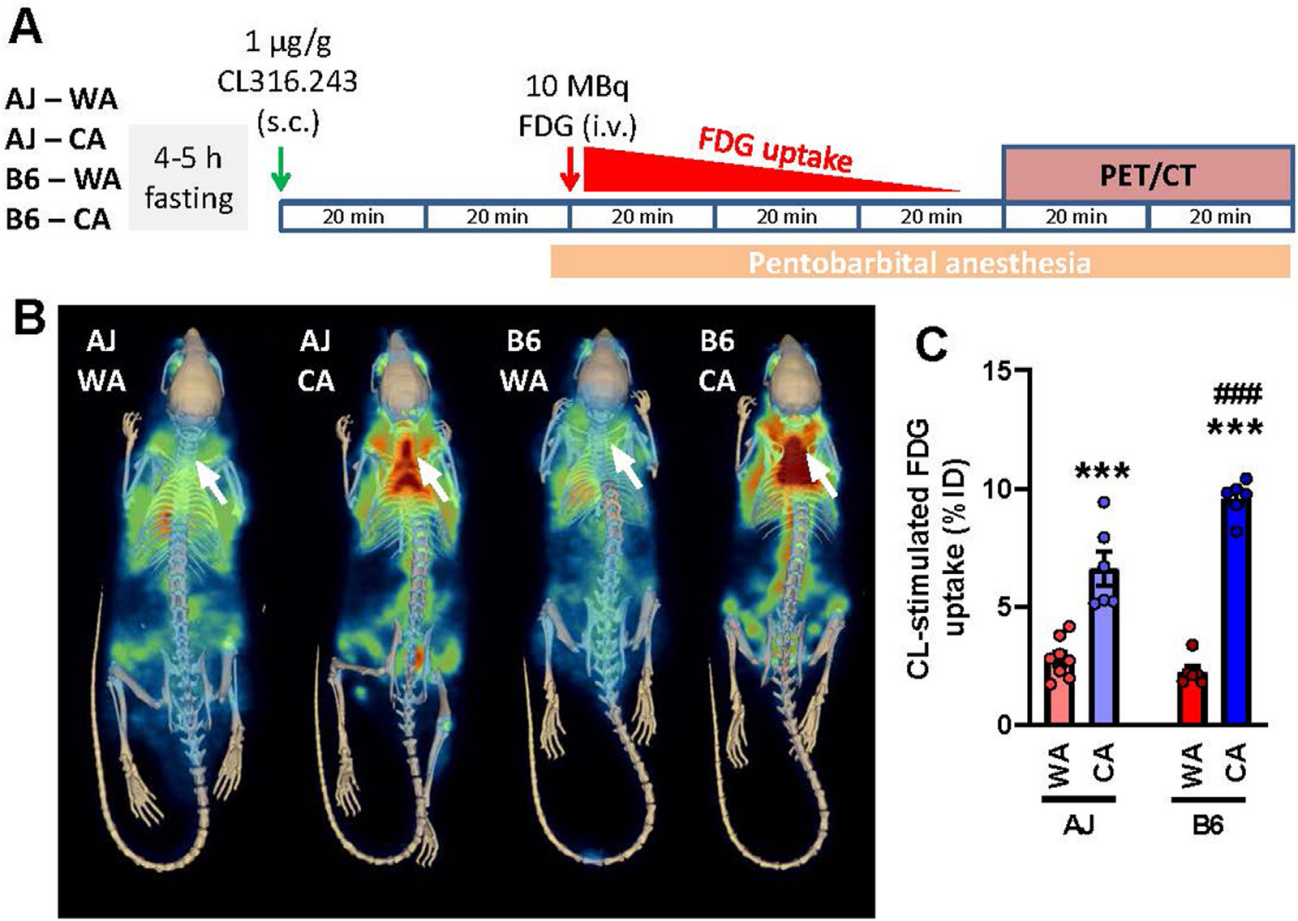
**CA results in higher induction of adrenergically-stimulated glucose uptake in iBAT of B6 compared with AJ mice**. *In situ* glucose uptake in iBAT was evaluated using PET/CT imaging. Both AJ and B6 mice acclimated to a thermoneutral temperature (30 °C; WA) or to cold (6 °C; CA) were used (*n* = 6–8). **(A)** Scheme of the experiment: fasted mice were first injected with CL316,243 (CL), 40 min later (to allow enough time to reach the maximal response to CL) anesthetized by pentobarbital, placed on a heating pad, and injected with ^18^F-fluorodeoxyglucose (FDG). **(B)** Representative PET/CT scans showing FDG accumulation in tissues of the whole mice after stimulation with CL. Arrows indicate iBAT location. **(C)** Quantification of FDG uptake in iBAT; data are means ± SEM; ∗∗∗*p* < 0.001 vs. the WA mice; ^###^*p* < 0.001 vs. the AJ mice (two-way ANOVA and Tukey's multiple comparison test). **(C)** Values for each data point can be found in S1 Data.

Next, to get a better insight into the adrenergic control of iBAT function, we focused on adrenergically induced glucose uptake in BAT since this uptake was shown to be independent on UCP1-mediated thermogenesis [73]. We measured CL-stimulated glucose uptake in iBAT *in situ* using FDG and PET/CT imaging (Figure 4A). In the WA mice of both strains, the PET signal in the iBAT location was undistinguishable from the surrounding tissues. In the CA mice, the signal increased, indicating recruitment of the capacity for adrenergically-stimulated glucose uptake. The effect of CA was stronger in B6 vs. AJ mice (∼4.7- vs. ∼2.2-fold stimulation, respectively), resulting in the highest FDG uptake in the B6-CA mice (Figure 4B,C). These results suggested that the lack of increase in whole-body metabolic rate in response to adrenergic agonist administration in the AJ-CA mice reflects a more general defect in the responsiveness of their BAT metabolism.

### 3.5 iBAT proteome – major effect of the acclimation temperature and more changes in response to CA in B6 vs. AJ mice

**Figure 5:**
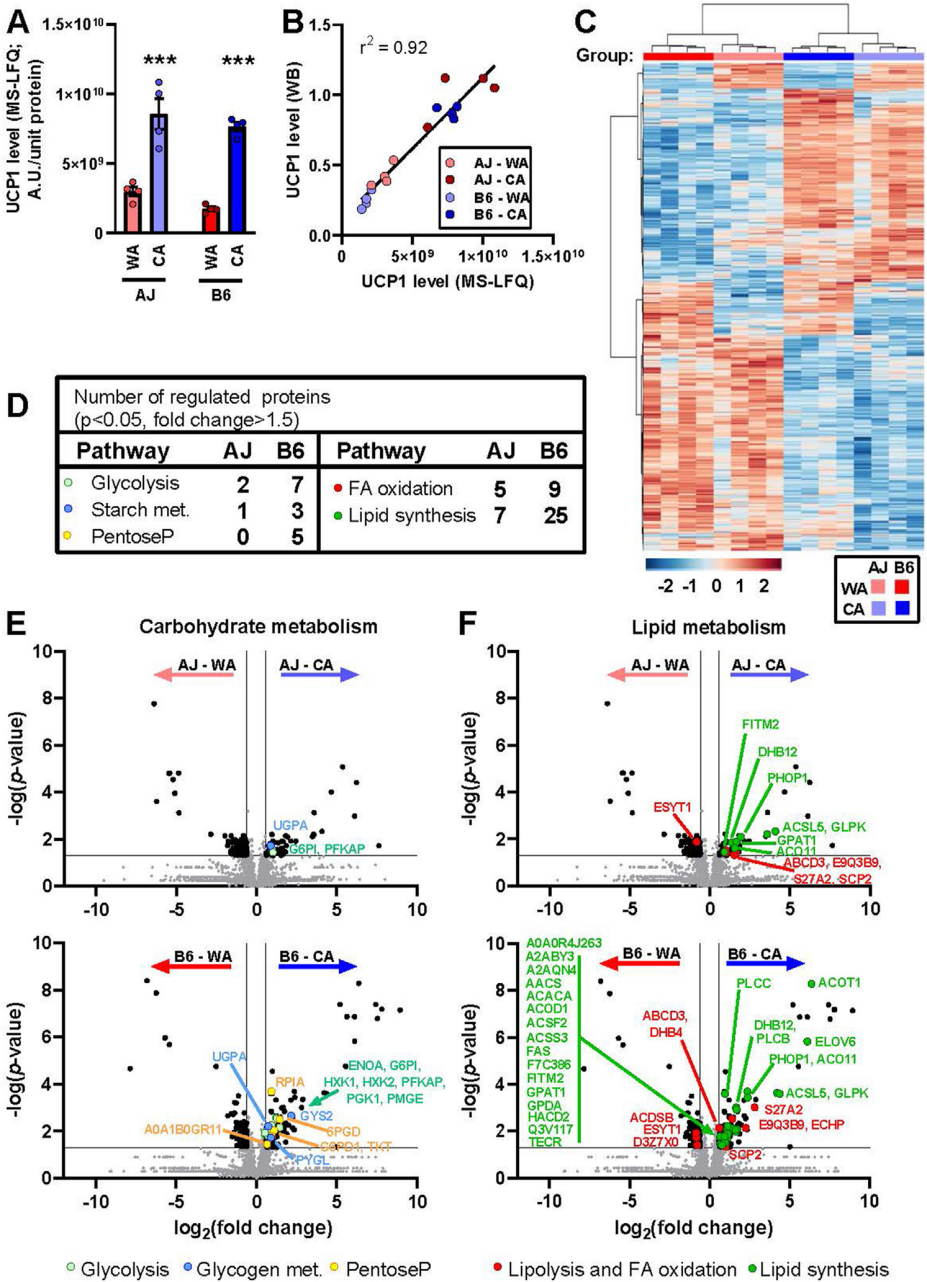
**Acclimation temperature dominates over the mouse strain in the effects on iBAT proteome**. Analyses of iBAT proteome of AJ and B6 mice acclimated to a thermoneutral temperature (30 °C; WA) or to cold (6 °C; CA) was performed using mass-spectrometry label-free quantification (MS-LFQ; *n* = 4). **(A)** Specific UCP1 content in iBAT homogenate; data are means ± SEM; ∗∗∗*p* < 0.001 vs. the WA mice (two-way ANOVA and Tukey's multiple comparison test). **(B)** Comparison of UCP1 quantification using MS-LFQ and Western blotting (WB); analysis performed as in Figure 2B. **(C)** Hierarchical clustering of proteins based on MS-LFQ intensity. Each column represents an individual animal (experimental group is indicated by colour code above the column), each row represents an individual protein. Only proteins differing significantly among the experimental groups (one-way ANOVA) were considered (i.e. 570 out of 3501 proteins detected). Both mice and proteins were automatically clustered using MetaboAnalyst (v 4.0 and 5.0) software [70] as indicated by dendrograms above and left of the plot. For the list of proteins, see S2 Data (sheet “BAT_ANOVA”). Hue represents the autoscaled t-test/ANOVA score. **(D, E, F)** Effect of temperature on the abundance of individual proteins in AJ and B6 mice. Total number of proteins/enzymes of selected metabolic pathways affected by cold in AJ and B6 mice (**D**). Volcano plots showing effects of temperature on proteins involved in glucose (**E**) and lipid metabolism (**F**). For the background data see S2 Data). For the effect of the strain on the above proteins, see S3 Fig. For the Volcano plot analysis of the quantitative composition of protein subunits of mitochondrial oxidative phosphorylation system, see S4 Fig. Volcano plots to demonstrate the difference in quantitative proteome composition between the WA- and the CA-mice, based on all 3501 proteins detected; S2 and S3 Data); plotted separately for AJ and B6 mice (upper and lower panels, respectively). Significantly regulated proteins (i.e. *p*-value <0.05; fold change >1.5) were (i) indicated by black dots (in AJ-WA vs. AJ-CA comparison: 48 proteins upregulated by CA and 85 proteins downregulated by CA; in B6-WA vs. B6-CA comparison: 140 proteins upregulated by CA and 93 proteins downregulated by CA; see S3 Data); (ii) ascribed to major metabolic pathways using KEGG database (see S3 Data); and (iii) color-coded according their involvement in carbohydrate (**E**) or lipid (**F**) metabolism. For the protein codes, see the entry name of UniProt database (used here without the name of the organism, e.g. UCP1 is originally UCP1_MOUSE; see S3 Data). (**A, B**) Values for each data point can be found in S1 Data.

As shown above, the relatively high cold endurance of AJ mice did not reflect adrenergically-controlled UCP1-mediated thermogenesis in BAT. However, NST in BAT might be activated in cold by another mechanism, independent of adrenergic stimulation. Therefore, in order to fully understand the role of BAT, label-free analysis of the whole iBAT proteome was performed in all groups of mice (*n* = 4). In total, 3501 proteins were detected (for list of proteins with Uniprot and Gene Ontology IDs and with values for all individual samples, see S2 Data). First, we verified (Figure 5A,B) the results of Western blot quantification of UCP1 in iBAT homogenates (Figure 2B–D). Indeed, strong positive correlation between the readouts of the two methods was found (Fig 5B).

Next, we performed hierarchical clustering of 570 proteins that were differentially expressed among the groups (*n* = 4; S2 Data, Fig 5C). At the first and the second level of hierarchy, all the mice were separated according to the acclimation temperature and the strain, respectively. Accordingly, pair comparisons of the whole iBAT proteome between experimental groups using Volcano plots revealed that more proteins were affected by the acclimation temperature (Figure 5D–F) than by the strain (S4A – C Fig). When the WA- and the CA-mice were compared within each strain, a higher number of regulated proteins were detected in B6 than in AJ mice (i.e. 238 vs. 133; Figure 5D–F; for a detailed list, see S3 Data). Thus, relatively strong induction by CA of enzymes engaged in glycolysis and glycogen metabolism was observed in B6 mice (Figure 5D,E), showing up to 4.5-fold increase with hexokinase 2 (**HXK2**) (S3 Data). This was in agreement with the relatively strong induction of iBAT glucose uptake by CL in the B6–CA mice (Fig 4). Also the levels of enzymes engaged in lipid metabolism were elevated by CA, with a stronger effect in B6 mice (Figure 5D,F), showing up to 7-fold increase in the level of very long-chain acyl-CoA synthetase (**S27A2**; S3 Data), which is involved in both lipid synthesis and FA degradation. In agreement with the previous results [71], the most pronounced effect of CA was induction of enzymes involved in FA synthesis (i.e. DNL), especially in B6 mice, documented the best by the levels of acyl-CoA thioesterase 1 (**ACOT1**) and FA elongase 6 (**ELOV6**) levels, respectively (Figure 5F, S3 Data). FA synthesis requires reduced NADPH provided by pentose phosphate pathway. Indeed, the abundance of enzymes of this pathway was elevated in B6-CA mice (Figure 5D,E). Glycerol kinase (**GLPK**) that is essential for TAG synthesis in BAT [5] was strongly induced by CA in both AJ and B6 mice (Fig 5F). Thus, both DNL and TAG synthesis helped to replenish TAG stores in adipocytes. In accordance with the induction of anabolic pathways by CA, levels of several protein subunits of the complexes of mitochondrial oxidative phosphorylation system (**OXPHOS**) that provides ATP were increased by CA, with a more pronounced effect in B6 mice (S5 Fig).

Taken together, the proteomics data documented an adaptive induction of iBAT metabolism in response to CA in B6 mice, which enhanced the capacity for adrenergically-regulated UCP1-mediated NST. In contrast, this induction was compromised in AJ mice.

### 3.6 Muscular activity (shivering) – no major difference between strains

**Figure 6:**
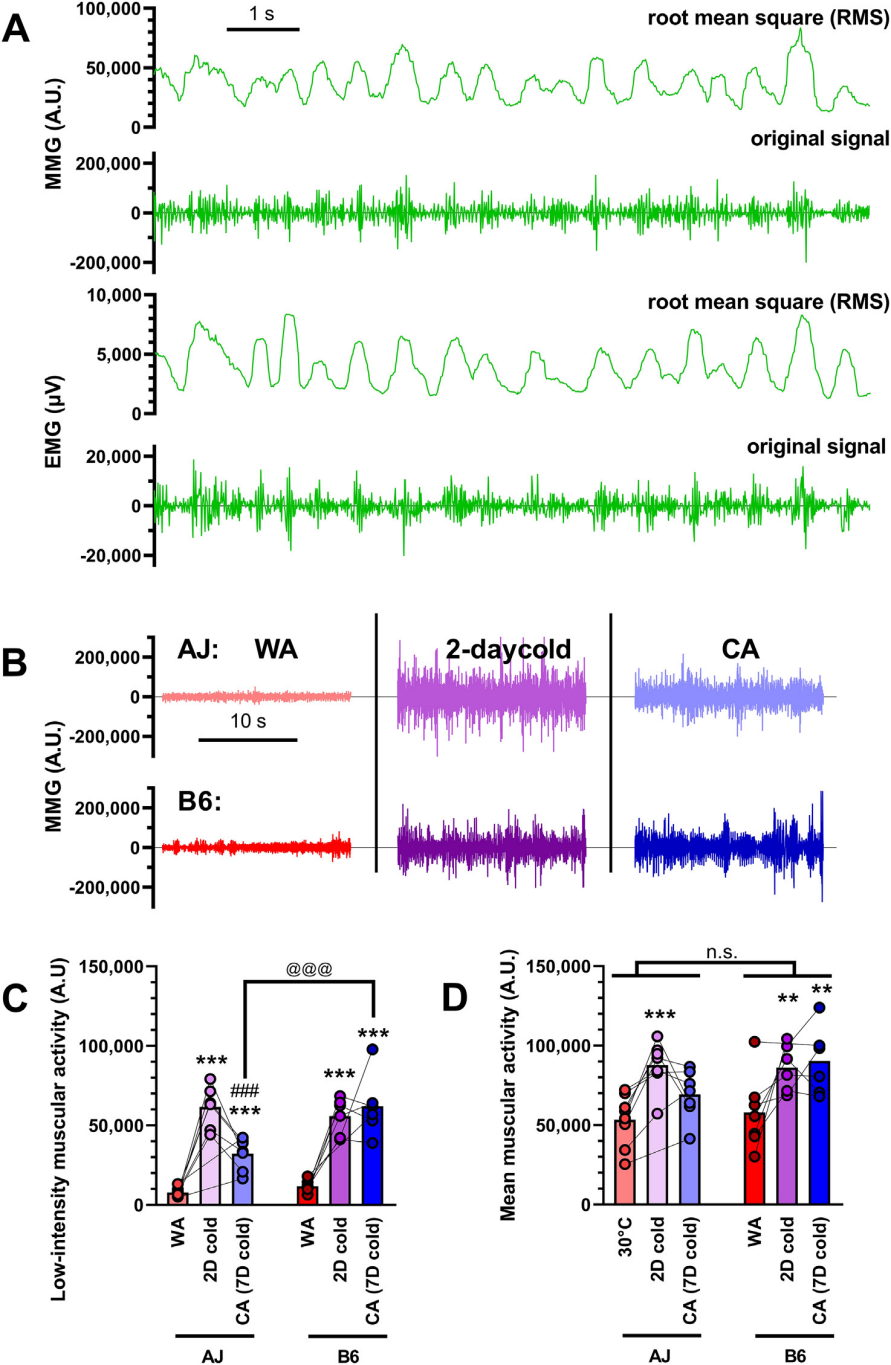
**Muscular activity (shivering) shows no major difference between strains.** EMG and MMG measurement of muscular activity were performed on AJ and B6 mice adapted to thermoneutral temperature (30 °C; WA) and consecutively exposed to cold (6 °C) for 2 and 7 days (CA) (*n* = 5–8). **(A)** Comparison of EMG and MMG signals from mice exposed acutely to cold showing both raw data and root mean square of these data. **(B)** Representative MMG measurement showing 20 s of low-intensity signal (i.e. without occurrence of physical activity) in each of the experimental conditions. **(C)** Quantification of low-intensity muscular activity (i.e. 15s-long signal out of 4 min MMG record with minimal or no physical activity). **(D)** Mean total muscular activity (i.e. including also muscle work). **(C**–**D)** ∗∗*p* < 0.01, ∗∗∗*p* < 0.001 vs. the respective WA group, ###*p* < 0.001 vs. the respective group after 2 days in cold (repeated measures mixed effect model (REML) and Tukey's multiple comparison test); @@@ *p* < 0.001 between the indicated groups (Sidak's multiple comparison test). Values for each data point can be found in S1 Data.

Next, in order to explain similar cold endurance of AJ and B6 mice in the face of vastly different capacity of adaptive NST in iBAT, we attempted to assess the role of shivering thermogenesis. In this respect, EMG is considered to be the gold standard for evaluation of the muscle activity [8] and is occasionally used in laboratory rodents [43,74,75]. More recently, with the development of accelerometers, MMG was introduced as a sensitive alternative to EMG [76]. To select the optimal technique for our measurements, we first compared EMG and MMG signals in mice exposed to acute cold (Figure 6A). Both techniques give signals with comparable resolution, which correspond closely to each other.

In contrast to most of the previous studies, we did not assess the thermogenic activity of mice immediately after implantation of the electrodes but several days later, allowing mice to recover after the surgery. This approach allowed us to avoid the potentially confounding effect of postsurgical stress and anaesthesia, which is known to inhibit thermoregulatory control, to increase inter-threshold range [77], and to influence subcutaneous blood flow, sympathetic outflow and skeletal muscle tone [78]. Due to this setting, we were also able to measure the individual mice repeatedly under various thermal conditions. In this experimental setup, the inter-individual variability of the EMG signals was largely increased, leading us to choose less invasive MMG technique, which provided more consistent result (Fig. 6B).

Thermogenesis, which results from muscle fibres contraction, includes a broad range of events [79] from physical work, through visually noticeable bursts of shivering, to subtle shivering microvibrations also called thermogenic muscle tonus [29,30], low-intensity muscular activity, resting mechanical activity, low–intensity activity, etc [76,80]. The thermogenic role of this low-intensity muscular activity was emphasized earlier, so we first analyzed this parameter (defined in [43] as lowest 15s-long signal out of 4 min record with minimal or no physical activity; Fig. 6C). In both AJ and B6 mice, the low-intensity muscular activity was negligible at thermoneutrality and largely increased after both 2 and 7 days of cold exposure (Fig 6B). Prolonged cold exposure should result in gradual recruitment of UCP1–mediated NST, leading to consequent drop in shivering activity [1,3,4,8,11]. We saw this effect in AJ mice between days 2 and 7 of cold exposure as a drop of low-intensity muscular activity, while B6 mice maintain high low-intensity muscular activity (Fig. 6C). As a result, AJ-CA mice did not show higher low-intensity muscular activity than B6-CA mice (rather the opposite), which disproves the possibility that higher level of shivering can explain the cold resistance of AJ mice.

Besides strictly defined low-intensity muscular activity, all muscle work in general (including physical activity, high-intensity shivering bursts etc.) produces heat and contributes to thermal balance of the organism. Therefore, we also analysed mean total muscular activity simply by calculating the mean MMG signal (Fig. 6D). In contrast to shivering only, this parameter was high already in WA animals, presumably reflecting high level of physical movements at thermoneutrality. Similarly to low-intensity muscular activity, the total mean muscular activity was also significantly increased after 2 days in cold and later tended to decrease back in AJ but not in B6 mice thermogenic muscle tonus [29,30] (Fig 6C). Thus, also total muscular activity is higher in B6-CA than in AJ-CA mice and cannot therefore explain the differences in cold endurance between the two genotypes.

### 3.7 Muscle proteome – major effect of the strain and more pronounced changes in response to CA in AJ vs. B6 mice

**Figure 7:**
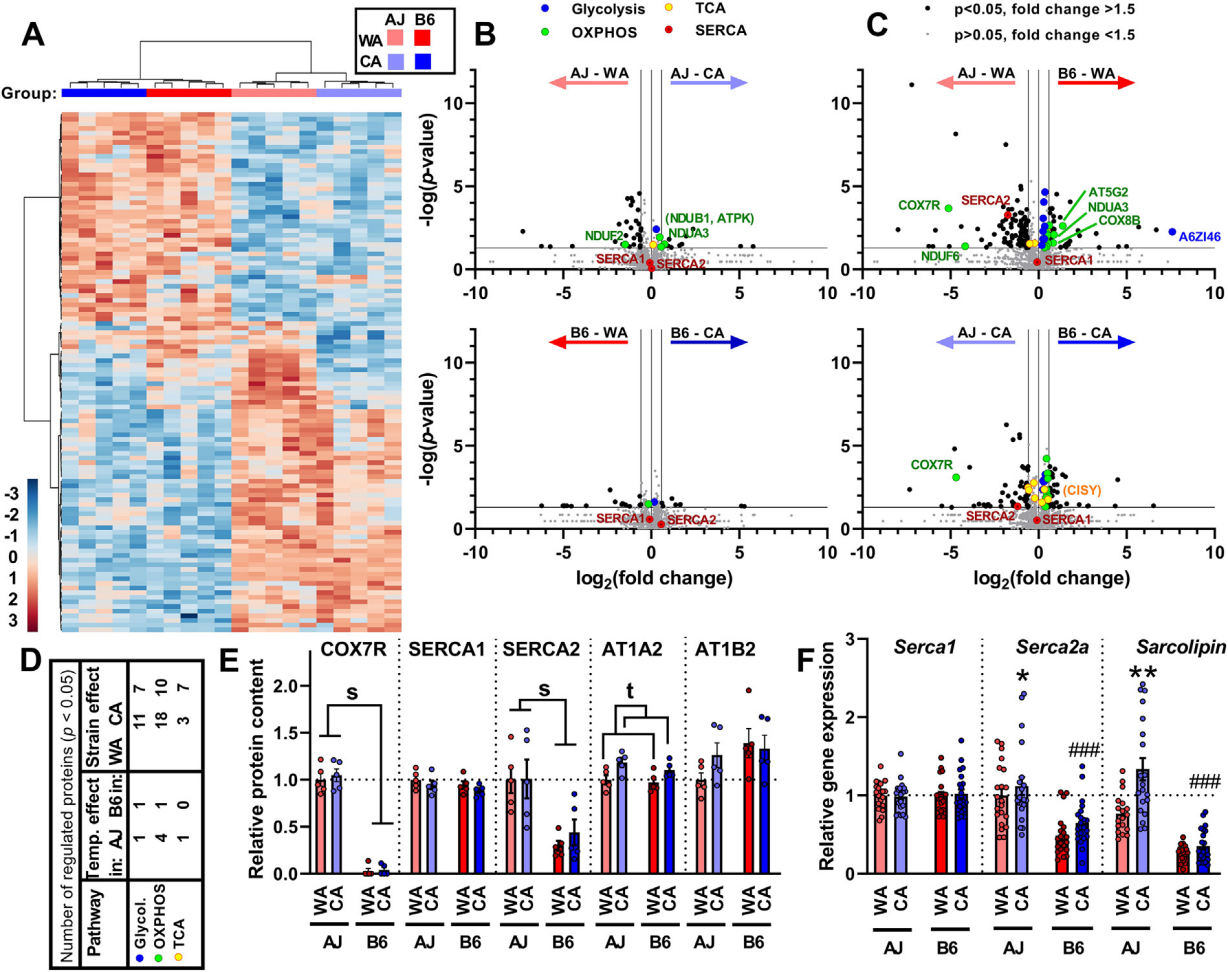
**Acclimation temperature exerts a weaker effect compared with the mouse strain on skeletal muscle proteome**. Analyses of gastrocnemius muscle proteome of AJ and B6 mice acclimated to a thermoneutral temperature (30 °C; WA) or to cold (6 °C; CA) was performed using mass-spectrometry label-free quantification (MS-LFQ; *n* = 4). **(A)** Hierarchical clustering of proteins based on MS-LFQ intensity. Each column represents an individual animal (experimental group is indicated by color code above the column), each row represents an individual protein. Only proteins differing significantly among the experimental groups (one-way ANOVA) were considered (i.e. 112 out of 1781 proteins detected in total). Both proteins and mice were automatically clustered using MetaboAnalyst (v 4.0 and 5.0) software [70] as indicated by dendrograms above and left of the plot. For the list of proteins, see S2 Data (sheet “GASTRO_ANOVA”). Hue represents the autoscaled t-test/ANOVA score. **(B, C, D)** Differences in proteome among the experimental groups. Volcano plots to demonstrate the difference in quantitative proteome composition between the WA- and the CA-mice (**B**) and between the AJ and B6 mice (**C**), based on all 1771 proteins detected; S2 and S3 Data). Significantly regulated proteins (i.e. raw *p*-value <0.05; fold change >1.5) were (i) indicated by black dots (in AJ-WA vs. AJ-CA comparison: 12 proteins upregulated by CA and 32 proteins downregulated by CA; in B6-WA vs. B6-CA comparison: 8 proteins upregulated by CA and 17 proteins downregulated by CA; in AJ-WA vs. B6-WA comparison: 100 proteins upregulated in AJ and 39 proteins upregulated in B6; in AJ-CA vs. B6-CA comparison: 56 proteins upregulated in AJ and 30 proteins upregulated in B6 see S3 Data); (ii) ascribed to major metabolic pathways using KEGG database (see S3 Data). The selected proteins are labelled by protein codes (for the protein codes, see the entry name of UniProt database - used here without the name of the organism, e.g. COX7R is originally COX7R_MOUSE; see S3 Data). Significantly regulated proteins mentioned in text but with fold change <1.5 are labelled by code in parentheses.). (**D**) Number of differentially regulated proteins/enzymes engaged in selected metabolic pathways (see B and C) in mice of both strains (see S3 Data). (**E**) Levels of selected proteins (see **B** and **C** and S3 Data; *n* = 4). (**F**) Expression of selected genes (measured using qPCR; data were normalized to a geometric mean of 2 housekeeping genes, see Materials and Methods). Data combined from 3 independent experiments; *n* = 18–21; data are means ± SEM; ∗*p* < 0.05 and ∗∗*p* < 0.01 vs. the WA mice; ###*p* < 0.001 vs. the AJ mice (two-way ANOVA and Tukey's multiple comparison test). (**E, F**) Values for each data point can be found in S1 Data.

The data above suggested induction of NST outside BAT in AJ-CA mice. In order to reveal whether NST in skeletal muscle could be involved, proteomic analysis was performed in gastrocnemius muscle, a representative muscle comprised of a mixture of type I, IIa, and IIb fibres. All four groups of mice were used for the analysis (*n* = 4). In total, 1771 proteins were detected (S2 Data). Hierarchical clustering of 112 proteins that were differently expressed among the groups (Figure 7A) revealed a separation of all the mice depending on the strain. At the second level of hierarchy, within each strain, all the animals separated according to the acclimation temperature. Thus, in contrast with iBAT (Fig 5A), a stronger effect of the strain than that of the acclimation temperature was observed.

In accordance with the above results, Volcano plots of the data revealed fewer differences in quantitative composition of the muscle proteome between the temperatures (Figure 7B,D) as compared with the genotype (Figure 7C,D). Frequent genotypic differences were detected in mitochondrial OXPHOS proteins, mostly a moderate upregulation in B6 mice, with more differences observed in the WA than the CA mice (Figure 7C,D). An important exception was a mitochondrial protein **COX7R** (product of *Cox7a2l* gene), also named ‘Supercomplex Assembly Factor I’ (**SCAFI**) [[81], [82], [83]]. Its levels were substantially higher in AJ than in B6 mice, in which the levels were extremely low (Figure 7C,E; and S6D Fig; see below and Discussion). Enzymes of carbohydrate metabolism showed elevated levels in B6 mice (see e.g. fructose-bisphosphate aldolase - **A6ZI46** in the WA mice; Figure 7C,D and S3 Data). The levels of several TCA cycle enzymes also showed strain-dependent modulation, being higher in AJ mice, especially in the CA animals (the inter-strain difference <1.4-fold for citrate synthase - **CISY**; Figure 7C,D), the canonical mitochondrial marker. When the WA and the CA mice were compared within each genotype (Fig 7B), a higher number of regulated proteins were detected in AJ compared to B6 mice (i.e. 44 vs. 25; for a detailed list, see S3 Data). The noticeable effect was a regulation of 4 OXPHOS proteins in AJ mice, which were mostly upregulated by CA (Figure 7B,D). This effect was maximally 1.7-fold (**NDUA3**; S6C Fig and S3 Data).

Next, we revisited our proteomic data with a focus on the candidate proteins involved in muscle NST, while also considering proteins that did not show significant differences in their abundance in the Volcano plots (Figure 7B,C). Regarding the futile DNL/FAox cycle [32], increase of acetyl-CoA carboxylase 2 (**ACACB**) and a trend for increase in the levels of both fatty acid synthase (**FAS**) and mitochondrial carnitine/acylcarnitine carrier protein (**MCAT**) levels in response to CA was found (S2 Data and S6A Fig), suggesting increased activity of DNL in the CA mice, independent of the genotype. However, none of the enzymes engaged in the transport of FA into mitochondria and their β-oxidation was affected by CA (S2 Data and S6B Fig).

Second, the role of futile SERCA activity induced by sarcolipin [8,9,35,36] was investigated. Levels of SERCA1 (in Figure 7E, and in S2 and S3 Data under UNIPROT entry name **AT2A1**) were similar in all the groups. However, levels of SERCA2 (in S2 and S3 Data as **AT2A2**) were higher in AJ than in B6 mice, independent of the acclimation temperature (Figure 7C,E). At the gene expression level, a similar pattern was observed, i.e. no differences among the groups with *Serca1* and higher expression of *Serca2a* in AJ vs. B6 mice. Moreover, *Serca2a* was (in AJ), or tended to be (in B6), upregulated in response to CA (Fig 7F). The efficiency of calcium pumping by SERCA is decreased, and hence energy dissipation during ATP hydrolysis increased, by the SERCA regulatory peptide sarcolipin. Since sarcolipin is a relatively short (31 amino acid) peptide, it was not detected using the label-free analysis of the muscle proteome. However, the qPCR revealed higher sarcolipin gene (***Sln***) expression in AJ vs. B6 mice. Importantly, *Sln* expression was upregulated ∼1.7-fold by CA in AJ but not in B6 mice (Fig 7F). Ca^2+^ cycling mediated by SERCA may therefore contribute to the CA-induced increase of UCP1-independent thermogenesis exclusively in AJ mice.

Eventually, we focused on Na^+^/K^+^-ATPase, which generates heat by maintaining Na^+^ and K^+^ electrochemical potential gradients across plasma membrane [25,26]. The levels of the catalytic AT1A2 (**Na**^**+**^**/K**^**+**^**-ATPase α2**) subunit, the major isoform expressed in skeletal muscle [84], were elevated in response to CA in mice of both genotypes. The levels of the non-catalytic AT1B2 (**Na**^**+**^**/K**^**+**^**-ATPase β2**) subunit were similar in all the groups (Fig 7E). These data suggested that Na^+^/K^+^-ATPase could contribute to cold-induced thermogenesis in skeletal muscles in both AJ and B6 mice.

### 3.8 Lipid oxidation, cytochrome *c* oxidase activity and changes in respiratory supercomplexes in skeletal muscle – stronger response to CA in AJ vs. B6 mice

Untargeted metabolite and lipid analysis of extracts prepared from gastrocnemius muscle detected 506 known analytes, mostly complex lipids (S4 Data). In accordance with the muscle proteome data (Fig 7), hierarchical clustering of 176 analytes differentially expressed among the groups revealed a stronger effect of the strain as compared to that of the acclimation temperature. Especially 17 acylcarnitines (out of 50 acylcarnitine species or their isobars that were detected; see S3 Table) contributed to the separation of the clusters (S7 Fig).

**Figure 8:**
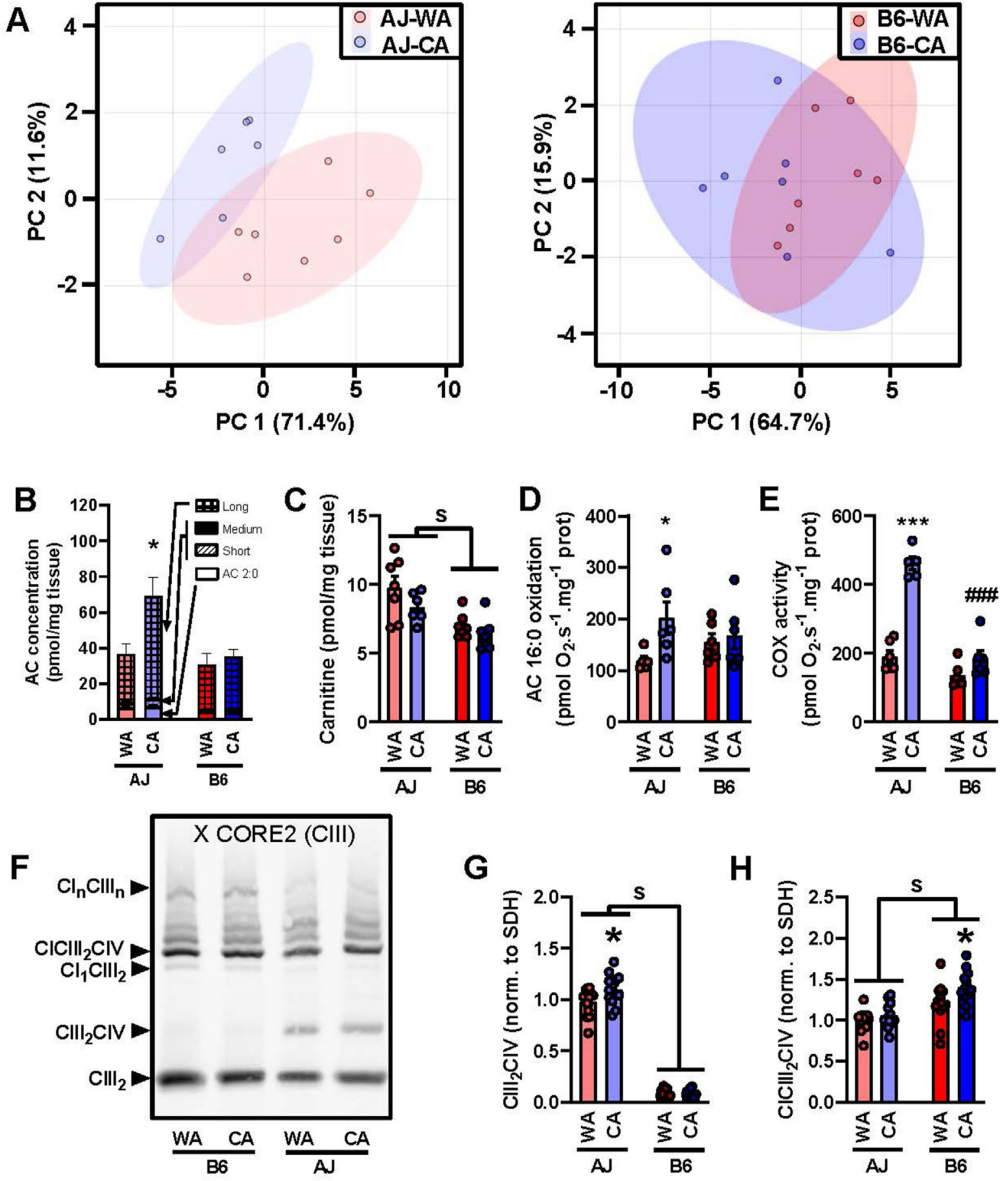
**CA results in higher induction of lipid catabolim and formation of Q-respirasome in skeletal muscle of AJ compared with B6 mice**. Measurements in gastrocnemius muscle extracts **(A**–**C)** and homogenates **(D**–**H)** prepared from AJ and B6 mice acclimated to a thermoneutral temperature (30 °C; WA) or to cold (6 °C; CA). (**A**) Principal component analysis of all acylcarnitine species in gastrocnemius muscle. **(B)** Cumulative concentrations of acylcarnitine (AC) species, which significantly differ in their abundances among the groups: acetylcarnitine (2:0), short-chain AC (3–7 carbons), medium-chain AC (8–15 carbons, very low abundances), long-chain AC (16 and more carbons) statistics performed on sum of all AC. **(C)** Concentration of carnitine (*n* = 6–7). **(D)** Oxidation of palmitoyl carnitine (*n* = 5–6). **(E)** Activity of COX (*n* = 5–6). **(F**–**H)** OXPHOS supercomplexes in digitonin solubilisates of gastrocnemius muscle analysed by blue native electrophoresis (*n* = 13–14; 7 mice in 2 technical replicates). Representative blot (**F**) and quantification of CIII_2_CIV (**G**) and CICIII_2_CIV (**H**). Data are means ± SEM; s indicates *p* < 0.05 effect of strain (two-way ANOVA); ∗*p* < 0.05 and ∗∗∗*p* < 0.001 vs. the WA mice, #*p* < 0.05 and ###*p* < 0.001 vs. the AJ mice (Tukey's multiple comparison test). For the source data in **A**, see S4 Data; for the source data in **B** and **C**, see S3 Table. **(B**–**H)** Values for each data point can be found in S1 Data.

Acylcarnitines represented major discriminating analytes. Notably, even-chain acylcarnitines, which arise from incomplete oxidation of FA, could serve as a complex biomarker of FA oxidation [[85], [86], [87]]. Using PCA of all acylcarnitine species detected, a separation between the WA and the CA mice was observed in AJ but not in B6 mice (Figure 8A). The even-chain acylcarnitines represented the majority of all acylcarnitines detected (S3 Table), while levels of more of them (18 vs. 10, respectively; ranging in length from C4 – C22) were increased in response to CA in AJ as compared with B6 mice (S3 Table). This was also illustrated by the sum of the levels for those with the long chain, which were induced in AJ mice (but not in B6 mice) by CA (Fig 8B). These data suggest relatively high *in vivo* adaptive increase in FA oxidation activity in the muscle of AJ mice in response to CA. Moreover, the concentration of carnitine was higher in AJ than in B6 mice, independent of the acclimation temperature (Fig 8C). Thus, AJ compared with B6 mice seemed to possess higher capacity for FA translocation to muscle mitochondria.

The above findings prompted us to directly assess the capacity for mitochondrial FAox in gastrocnemius muscle (S8 Fig). Respirometry of substrates oxidation in muscle homogenates revealed ∼1.7-fold increase in the activity of palmitoyl carnitine in response to CA in AJ but no activity change in B6 mice (Fig 8D). The activity of COX, the terminal enzyme of the mitochondrial respiratory chain, was similar in all the groups, except for a much higher activity in the AJ-CA mice, which showed a ∼2.5-fold increase over their AJ-WA counterparts (Fig 8E). However, the differences in the biochemical activities did not correspond to the levels of the enzymes involved in FAox or the levels of COX subunits, respectively (S2 Data and S6B and S6D Fig), suggesting a higher level of control of the activities of these enzymes.

The muscles of AJ and B6 mice differed significantly in the levels of COX7R/SCAFI [[81], [82], [83]] (Figure 7C,E; and S5D), a factor that affects CIII_2_CIV supercomplex formation [[88], [89], [90]]. Therefore, we examined whether differences in the respiratory chain supercomplexes organisation could underlie the strain-specific induction of COX activity by CA in gastrocnemius muscle. For this purpose, we performed blue native gel electrophoresis of digitonin solubilized muscle samples (Fig 8F). Immunodetection of respiratory chain complex III showed marked differences in the content of the CIII_2_CIV supercomplex (Q-respirasome), which was barely detectable in B6 mice (in both WA and CA groups) but it was present in much higher amounts in the AJ-WA mice (∼9-fold compared to the B6-WA mice) and was further increased in the AJ-CA mice (Fig 8G). In contrast, the content of the CICIII_2_CIV supercomplex (N-respirasome) was significantly lower in AJ compared to B6 mice (∼20%) and even increased in the B6-CA mice compared to their B6-WA counterparts (Fig 8H). Thus, the observed increase in COX activity by CA in AJ mice was associated with, and likely supported by, a high Q-respirasome or increased Q/N respirasome ratio in AJ vs. B6 mice.

Collectively, the above results demonstrate strain-specific regulation of oxidative lipid metabolism in skeletal muscle, with increased FAox capacity in response to CA observed only in AJ mice.

## 5 Discussion

For a long time, higher UCP1-dependent thermogenesis was thought to be the reason for obesity resistance and cold endurance of AJ mice, in contrast to strains such as B6 [18]. It was thus surprising to find out that constitutively high cold endurance of AJ mice was associated with UCP1-independent thermogenesis, while adaptive thermogenesis in BAT was impaired. On the contrary, cold endurance of B6 mice depended on the adaptive induction of UCP1-mediated thermogenesis by CA. These results are not in agreement with our original hypothesis that higher activation of TAG/FA cycling associated with FA release from WAT in response to CA in AJ compared to B6 mice reflected combustion of FA for thermogenesis in BAT [52]. Instead, the results document that the higher activation of the FA release in AJ mice is needed for UCP1-independent thermogenesis.

The unexpected impairment of the CA-inducible thermogenesis in BAT of AJ mice was evidenced by (i) whole-body metabolic response to adrenergic agonists NE and CL, (ii) CL-stimulated glucose uptake in iBAT, and (iii) changes in cold adaptive iBAT proteome. Increase in *Ucp1* expression in rpWAT by CA was more pronounced in AJ mice, in agreement with the previous findings [15]. However, total UCP1 content in both iBAT and iWAT, the two major UCP1-containing fat depots, was elevated to a similar level in mice of the two genotypes. Also UCP1 activity and its control by GDP in isolated iBAT mitochondria were similar in both AJ and B6 mice. The impairment of the adaptive thermogenesis in iBAT of AJ mice may result from a defect in the adrenergic signalling pathway downstream of adenylate cyclase, which showed similar activation by both NE and β_3_-agonist in mice of the two genotypes [18]. This would result in impaired activation of BAT metabolism, including lipolysis, UCP1 protonophoric (i.e. thermogenic) activity, as well as glucose uptake in adipocytes as observed here. However, detailed characterization of mechanism behind the impairment of adaptive NST in BAT remains out of the scope of this study.

The compromised induction of BAT thermogenic activity in AJ-CA mice was not compensated either by classical muscular shivering activity, which was rather higher in B6-CA than in AJ-CA mice, or by physical activity. After acute cold exposure, the physical activity tended to be higher in B6 mice than in AJ mice. When we analyzed the mean total MMG signal, which should include also physical activity, AJ-CA and B6-CA mice did not significantly differ. Also that AJ compared with B6 mice defended lower T_b_ had probably no major effect on cold-induced thermogenesis. Indeed, under similar experimental conditions as here, the cost of keeping T_b_ higher by 1 °C at ambient temperature of 4 °C was 4.4% of total energy expenditure [60]. Thus, the effect of the small difference (<1 °C) in the T_b_ set point between AJ and B6 mice on cold-induced energy expenditure would be relatively small, compared with the ∼4 – 5-fold difference of energy expenditure measured at 5 °C and 33 °C, respectively, independent of the strain. Moreover, both thermal insulation and heat conductance were similar in mice of both strains, and could not explain the compromised induction of adrenergically mediated thermogenesis in AJ mice. Collectively, these results prompted us to seek for an alternative UCP1-independent NST, which could explain the phenotype of A/J mice.

Indeed, our results document adaptive induction of lipid catabolism in response to CA, independent of UCP1, observed only in AJ mice, resulting probably in large from NST in skeletal muscle. This was in agreement with leftward shift in PRCF of RQ values observed at thermoneutral conditions, indicating a change in fuel partitioning in skeletal muscle, which is a major determinant of resting energy expenditure [24]. The involvement of muscle was directly evidenced by (i) changes in the level of mitochondrial citrate synthase and several OXPHOS proteins – except for COX (see below); (ii) changes in muscle acylcarnitine profile; and (iii) increase of specific activity of both palmitoyl carnitine oxidation and COX in muscle homogenates. In spite of the CA-induced lipid catabolism in skeletal muscle and enhanced oxidative capacity of the muscle, energy expenditure in acutely cold-exposed AJ mice was not affected by CA. These data suggest that unmasking of the adaptive induction of energy expenditure, i.e. NST, in the muscle by CA would require an even stronger cold stress, which could not be applied for technical reasons here (see Figure 1C–D and [58]). In fact, in B6 mice under the above-mentioned experimental conditions, only a marginal induction of adaptive NST could be detected, in spite of the pronounced induction of the capacity for UCP1-mediated NST in BAT (compare adrenergically-induced whole body metabolism in Fig 3 and UCP1 content in adipose tissue in Fig 2).

Increase of FAox activity by CA in AJ mice correlated with specific activity of COX, which increased ∼2.5-fold. Interestingly, this large increase was not accompanied by increase in protein content of individual COX subunits. This is in agreement with complex posttranslational control of the activity of this terminal enzyme of mitochondrial respiratory chain, which is required for the fine-tuning of energy supply to support various cellular functions. Indeed, COX activity depends on enzyme isoforms, binding of ligands, or conformational changes of the enzyme (reviewed in [91,92]). Importantly, rate of mitochondrial respiration may also be controlled at the level of energy dissipation occurring at COX itself [28].

Recent studies suggest that COX activity could also reflect its assembly in respiratory supercomplexes, which depends on the presence of COX7R/SCAFI, an isoform of COX7A protein subunit [[88], [89], [90]]. Mice showed strain-dependent differences in expression of two variants of COX7R/SCAFI protein, composed of 111 and 113 amino acids, respectively. Only the long, functional variant was present in CBA, 129sv, NZB, and CD1 mice, whereas C57BL/6 J and Balb/cJ mice were homozygous for the short, non-functional variant [81,82]. In accordance with these data, we found very low levels of COX7R/SCAFI protein and of CIII_2_CIV respiratory supercomplexes in the muscle of B6 compared to AJ mice thus confirming that COX7R/SCAFI is essential for Q-respirasome (CIII_2_CIV) formation. In contrast, N-respirasome supercomplexes (CICIII_2_CIV, reviewed in [88]) can assemble also with COX7A2 or COX7A1 subunits [27,93] and N-respirasome structural and functional organisation depends on prevalent COX7A isoform [27]. COX7R/SCAFI associated formation of Q-respirasome even led to a decrease in the content of N-respirasome in muscle of AJ compared to B6, which could improve oxidation of flavoprotein-dependent substrates and accordingly, the utilization of FA [82,88]. Since activity of COX is increased one order of magnitude when embedded in respirasome [89], even a small but significant increase in the level of the CIII_2_CIV supercomplex observed in AJ mice in response to CA could lead to the much larger increase in the COX activity (see above). Moreover, formation of SCAFI-containing respirasomes may decrease proton pumping efficiency and could thus have thermogenic effect *per se* [27]. Previous studies in mice demonstrated possible metabolic role of COX7R/SCAFI and its importance in modulation of muscle exercise performance [83,89], fat accumulation [83], body growth and fertility [94], or blood glucose levels [95]. Finally, the recent study [93] demonstrated that reconstitution of functional COX7R/SCAFI in B6 mice increased both energy expenditure and lean mass of the animals, and that polymorphism in COX7R/SCAFI gene was linked with adiposity and cardiorespiratory fitness in humans. Overall, the data indicate that expression of COX7R/SCAFI may support the strain-specific adaptive induction of NST by CA in skeletal muscle.

In any case, increase in the activity of mitochondrial COX in skeletal muscle of A/J mice in response to CA represents a surrogate marker of the induction of NST capacity. It may reflect either uncoupling of oxidative phosphorylation in muscle mitochondria (see above) or increased ATP formation, which is required for other metabolic pathways engaged in energy dissipation. Our data point namely to substrate cycling between DNL and lipid oxidation [[31], [32], [33], [34]], Na^+^/K^+^-ATPase [25,26], and uncoupling of SERCA activity by sarcolipin, with its firmly established role in muscle NST [8,9,[35], [36], [37]]. The last mechanism could play a major role and explain differential induction of the capacity of muscle NST by CA in mice of the two genotypes. Levels of SERCA2a and expression of its gene were higher in AJ mice, independent of the acclimation temperature. This was corroborated by a higher expression of *Sln*, and namely its induction by CA in AJ mice. Previous studies using gastrocnemius muscle of B6 mice demonstrated (i) a stimulation of SERCA2a expression by leptin, possibly through the thyroid axis, while leptin caused a shift in the substrate use from carbohydrates to fat [31], and (ii) an increase in SERCA2a and sarcolipin protein content by cold acclimation, while SERCA1a levels were decreased [41]. Only oxidative muscles rich in type I fibres but not glycolytic muscles appeared to have the capacity for sarcolipin-mediated SERCA uncoupling [9,31,96]. In AJ mice, we have also observed an induction of this mechanism by CA in gastrocnemius but not in tibialis muscle, in accordance with its higher type I fibres content (not shown). Collectively, the above data suggest that the mechanism of NST mediated by SERCA in skeletal muscle could be induced in mice with different genetic backgrounds, while AJ are more susceptible to the induction than B6 mice.

Our results are not in agreement with the view that only the capacity of UCP1-mediated thermogenesis could be adaptively increased in response to cold or diet [14,43]. Our data support the theory coined by L. P. Kozak two decades ago [10] (see also [6,15]) that UCP1-independent thermogenesis could be activated, in an adaptive manner, in the absence of BAT and provide protection against both cold and obesity. In these lines, it was also hypothesised that BAT could heat the body more effectively compared to muscle [6,9] at least in part due to its anatomic location [4]. The higher metabolic cost associated with muscle NST may also require different control mechanisms which trigger it. BAT thermogenesis is strictly activated during (i) periods of cold exposure or (ii) when animals are eating, i.e. during the dark phase of the day in mice [14]. On the other hand, muscle NST might be under a looser and less flexible control. When activated, it could dissipate energy partially independent of actual energy intake. We have previously shown that weaning AJ mice onto a high-fat diet stimulates oxygen consumption in oxidative muscle by elevating metabolic rate during the light phase of the day, which was not observed in B6 mice [33]. Similar strain-specific differences in the effect of high-fat feeding on energy expenditure were also observed in adult AJ and B6 mice (our unpublished results). Moreover, overexpression of sarcolipin in muscle rendered mice resistant to high-fat diet-induced obesity while oxygen consumption was elevated during both light and dark period of the day, independent of physical activity (Figure 3 of [40]).

Meals with high content of fat are obesogenic, due to low energetic cost of nutrient storage and low potency of fat intake to promote fat oxidation [97]. Human studies suggest that the lipid oxidation capacity may underlie susceptibility to obesity [46,98]. We show here that depending on the genetic background and reflecting propensity to obesity, mice may be predisposed for lipid oxidation in the muscle by adaptation to cold. Thus, also in humans, living in cold environment may induce NST in skeletal muscle, depending on the genetic setup of each individual. This could provide protection against cold and compromise development of obesity [[12], [13], [14]]. Characterization of the complex interplay of genes engaged in control of energy expenditure represents a challenge of a major practical significance with respect to the prevention and treatment of obesity and associated diseases. The role of various genes in the inter-individual differences in muscle thermogenesis in humans is becoming recognized only recently [29].

Our results suggest that AJ mice represent a model for characterizing UCP1-independent mechanisms of NST [2,[5], [6], [7]] outside BAT and their physiological role. Reflecting the relatively low thermogenic activity of their BAT, these mice may provide a better model of the situation in humans compared with B6 and mixed genetic background mice, used in most of the previous studies in this field with the focus on the role of UCP1 [14,43], SERCA-sarcolipin [9,38,42] or other mechanisms [7,10,22,23,31,36,50] in NST. To get further insight into the mechanisms engaged in NST and the role of genetic background [[15], [100]], several inbred strains of mice differing in susceptibility to dietary obesity [99] should be used in future studies.

In conclusion, we have shown here that organ-specific contribution to NST in mice depends on genetic background of the animals. The adaptive increase in the capacity for NST in skeletal muscle, in the face of impaired capacity for NST in BAT, was associated with resistance to obesity. Thus, the capacity for NST outside BAT, unmasked by CA and contributing to cold endurance, could also affect propensity to obesity. Obesity-prone phenotype could be caused by insufficient capacity for NST in skeletal muscle rather than in BAT.

## Author contributions

Conceptualization: JK, KB, MR, PJ, PZ. Formal analysis: LL, PZ. Funding acquisition: JH, JK, PJ, TM. Investigation: AR, JF, JO, KA, KB, MV, OH, PJ, PZ, SS, TC, TM, ZD. Project administration: PJ, KB. Supervision: JK. Validation: JH, KB, MV, PJ, PZ, TC, TM. Visualization: PZ, PJ, KB; Writing – original draft: PJ, PZ, JK. Writing – review & editing: all authors.

## Data Availability

Data will be made available on request.
