# Supplementary material for: Impairment of adrenergically-regulated thermogenesis in brown fat of obesity-resistant mice is compensated by non-shivering thermogenesis in skeletal muscle
Source: Mol Metab. 2023 Jan 30;69:101683. doi: 10.1016/j.molmet.2023.101683 (PMC9922683; doi:10.1016/j.molmet.2023.101683)
Supplement: Multimedia component 1 [file mmc1.docx]

**Supporting information**

***Content***

Supplementary methods (+ references)……………………………………………………………………………………….. p. 1

Legend to Supplementary Data files (Supplementary Data attached as .xls files)…………………………. p. 3

Supplementary tables S1 – S4 ……………………………………………………………………………………………………... p. 4

Supplementary figures S1 – S7 …………………………………………………………………………………………………….. p. 9

***Supplementary methods***

*Sample extraction.* Muscle samples (20 mg) were homogenized with 275 µL methanol containing internal standards (PE 17:0/17:0, PG 17:0/17:0, LPC 17:1, sphingosine d17:1, Cer d18:1/17:0, SM d18:1/17:0, PC 15:0/18:1-d7, cholesterol-d7, TG 17:0/17:1/17:0-d5, DG 12:0/12:0/0:0, DG 18:1/2:0/0:0, LPE 17:1, oleic acid-d9, PI 15:0/18:1-d7, MG 17:0/0:0/0:0, PS 17:0/17:0, HexCer d18:1/17:0, DG 18:1/0:0/18:1-d5, TG 20:0/20:1/20:0-d5, LPG 17:1, LPS 17:1, cardiolipin 16:0/16:0/16:0/16:0) and 275 µL 10% methanol containing internal standards (caffeine-d9, acetylcholine-d4, creatinine-d3, choline-d9, TMAO-d9, N-methylnicotinamide-d4, betaine-d9, butyrobetaine-d9, creatine-d3, cotinine-d3, glucose-d7, succinic acid-d4, metformin-d6, alanine-d4, arginine-d7, aspartic acid-d3, citrulline-d2, glutamic acid-d5, glycine-13C2-15N, leucine-d3, methionine-d3, ornithine-d6, phenylalanine-d5, proline-d7, tyrosine-d4, valine-d8, carnitine-d9, C2-carnitine-d3, C3-carnitine-d3, C4-carnitine-d3, C5-carnitine-d9, C5DC-carnitine-d6, C6-carnitine-d3, C8-carnitine-d3, C10-carnitine-d3, C12-carnitine-d3, C14-carnitine-d3, C16-carnitine-d3, C18-carnitine-d3) for 1.5 min using a grinder (MM400, Retsch, Germany). Then, 1 mL of MTBE with internal standard (CE 22:1) was added, the tubes were shaken for 1 min and centrifuge at 16,000 rpm for 5 min.

*LC-MS-based lipidomics.* The LC-MS system consisted of a Vanquish UHPLC System (Thermo Fisher Scientific, Bremen, Germany) coupled to a Q Exactive Plus mass spectrometer (Thermo Fisher Scientific, Bremen, Germany). Lipidomic profiling was used for the analysis of high-chain acylcarnitines (ACar 13:0–22:6) and other complex lipids. Specifically, 100 µL of the upper organic phase was collected, evaporated, and resuspended using 100 µL 80% methanol with internal standard (CUDA), shaken for 30 s, centrifuged at 16,000 rpm for 5 min, and used for LC-MS analysis.

Lipids were separated on an Acquity UPLC BEH C18 column (50 × 2.1 mm; 1.7 μm) coupled to an Acquity UPLC BEH C18 VanGuard pre-column (5 × 2.1 mm; 1.7 μm) (Waters, Milford, MA, USA). The column was maintained at 65°C at a flow-rate of 0.6 mL/min. The mobile phase consisted of (A) 60:40 (v/v) acetonitrile:water with ammonium formate (10 mM) and formic acid (0.1%) and (B) 90:10:0.1 (v/v/v) isopropanol:acetonitrile:water with ammonium formate (10 mM) and formic acid (0.1%). Separation was conducted under the following gradient: 0 min 15% (B); 0–1 min 30% (B); 1–1.3 min from 30% to 48% (B); 1.3–5.5 min from 48% to 82% (B); 5.5–5.8 min from 82% to 99% (B); 5.8–6 min 99% (B); 6–6.1 min from 99% to 15% (B); 6.1–7.5 min 15% (B). A sample volume of 2 μL was used for the injection. The sample temperature was maintained at 4°C.

The ESI source and MS parameters were: sheath gas pressure, 60 arbitrary units; aux gas flow, 25 arbitrary units; sweep gas flow, 2 arbitrary units; spray voltage, 3.6 kV; capillary temperature, 300°C; aux gas heater temperature, 370°C; MS1 mass range, m/z 200–1700; MS1 resolving power, 35,000 FWHM (m/z 200); number of data-dependent scans per cycle, 3; MS/MS resolving power, 17,500 FWHM (m/z 200); normalized collision energy, 20%.

*LC-MS-based metabolomics.* Metabolomic profiling was used for the analysis of carnitine, short-chain acylcarnitines (ACar 2:0–12:0), amino acids, and other polar metabolites. An aliquot of 70 µL of the bottom aqueous phase was collected, evaporated, resuspended in 70 µL of an acetonitrile/water (4:1, v/v) mixture with internal standards (CUDA and Val-Tyr-Val), shaken for 30 s, centrifuged at 16,000 rpm for 5 min and used for LC-MS analysis.

Metabolites were separated on an Acquity UPLC BEH Amide column (50 × 2.1 mm; 1.7 μm) coupled to an Acquity UPLC BEH Amide VanGuard pre-column (5 × 2.1 mm; 1.7 μm) (Waters, Milford, MA, USA). The column was maintained at 45°C at a flow-rate of 0.4 mL/min. The mobile phase consisted of (A) water with ammonium formate (10 mM) and formic acid (0.125%) and (B) acetonitrile:water (95/5) with ammonium formate (10 mM) and formic acid (0.125%). Separation was conducted under the following gradient: 0 min 100% (B); 0–1 min 100% (B); 1–3.9 min from 100% to 70% (B); 3.9–5.1 min from 70% to 30% (B); 5.1–6.4 min from 30% to 100% (B); 6.4–8.0 min 100% (B). A sample volume of 0.5 μL was used for the injection. The sample temperature was maintained at 4°C.

The ESI source and MS parameters were: sheath gas pressure, 50 arbitrary units; aux gas flow, 13 arbitrary units; sweep gas flow, 3 arbitrary units; spray voltage, 3.6 kV; capillary temperature, 260°C; aux gas heater temperature, 425°C; MS1 mass range, m/z 60–900; MS1 resolving power, 35,000 FWHM (m/z 200); number of data-dependent scans per cycle, 3; MS/MS resolving power, 17,500 FWHM (m/z 200); normalized collision energy, 20-30-40%.

*Quality control.* Quality control was assured by (i) randomization of the actual samples within the sequence, (ii) injection of quality control (QC) pool samples at the beginning and the end of the sequence and between each 10 actual samples, (iii) analysis of procedure blanks, (iv) serial dilution of QC sample (0, 1/16, 1/8, 1/4, 1/2, 1), (v) checking the peak shape and the intensity of internal standards spiked during the extraction and the internal standard added prior to injection.

*Data processing.* LC-MS data from lipidomic and metabolomic profiling were processed through MS-DIAL v. 3.80 software. Metabolites were annotated using in-house retention time–m/z library and using MS/MS libraries available from commercial and open sources (NIST17, MassBank, MoNA). Acylcarnitines were annotated using LipidBlast in-built in MS-DIAL (1). Raw data were filtered using blank samples, serial dilution samples, and QC pool samples with relative standard deviation (RSD) <30%, and then normalized using locally estimated scatterplot smoothing (LOESS) approach through QC pool samples injected regularly between 10 actual samples. Internal standards spiked during the extraction were used to calculate the concentration of carnitine, acylcarnitines, and amino acids (pmol/mg) in analyzed samples.

***References***

1. Tsugawa H, Cajka T, Kind T, Ma Y, Higgins B, Ikeda K, et al. MS-DIAL: data-independent MS/MS deconvolution for comprehensive metabolome analysis. *Nat Methods.* 2015;12(6):523-6.

***Supplementary data files***

**S1 Data. Underlying data for main figures and supporting figures.** Excel spreadsheet containing,

in separate sheets, the underlying numerical data and descriptive statistical analysis for individual Figs. 2-8, S2, and S5.

**S2 Data. Proteomic data of iBAT and gastrocnemius muscle – all data.** Related to Figs 5 and 7.

Sheets **“BAT_proteomics”** and **“GASTRO_proteomics”** contain lists of all identified proteins in each tissue (3501 proteins in iBAT, with 2527 of them detected in at least one mouse in each group; and 1771 proteins in gastrocnemius muscle, with 1642 of them detected in at least one mouse in each group) ordered according to UNIPROT ID and annotated by Gene Entrez ID, KO number, and Gene Ontology ID. Individual mice are ordered in columns and annotated using experimental group (AJ-WA, AJ-CA, B6-WA, B6-CA) and unique sample code (P100-P115 for iBAT proteomics, and P116-P135 for proteomics in gastrocnemius muscle). These data are used for multivariate analyses of proteom of the tissues (S3 Data, Fig 5, Fig 7, S2 Fig and S3 Fig). Sheets **“BAT_ANOVA”** and **“GASTRO_ANOVA”** contain proteins with different levels among the groups according to analysis of variance (one-way ANOVA; 570 proteins in iBAT, and 112 proteins in gastrocnemius muscle). Both *p*-value (“p_raw” column) and *p*-value adjusted to false discovery rate (“p_FDR” column) are shown. Tukey’s HSD (honest significance test) was used to identify the individual pairs of significantly different groups. The proteins are ordered according to the *p*-value. Levels of these selected proteins were plotted in heat maps in Fig 5C and Fig 7A. For further details see sheet “READ ME”.

**S3 Data. Proteomic data of iBAT and gastrocnemius muscle used for volcano plots.** Related to Fig 5, Fig 7, S2 Fig and S3 Fig. The individual sheets contain the pair comparisons of experimental groups using Student’s *t*-test. For each protein, fold change between the groups (“FC”), raw p-value (“p_raw”) and *p-*value adjusted to FDR (“p_FDR”; false discovery rate) are listed, as well as AXIS x (-log2(FC)) and AXIS y (-log10(p_raw or p_FDR)), which were used in Fig 5D-E and Fig 7B and 7C (p_FDR was used in the case of iBAT, p_raw was used in the case of gastrocnemius muscle). The proteins differently regulated between at least two groups were ascribed to major metabolic pathways using KEGG database; pathways containing several (>5) differently regulated proteins were identified (glycolysis, pentose phosphate pathway, starch metabolism, lipid synthesis and fatty acid oxidation, mitochondrial proteins of respiratory chain in iBAT; glycolysis, starch metabolism, tricarboxylic acid cycle, and oxidative phosphorylation in gastrocnemius muscle. In the individual sheets of this Data file, column “KEGG pathway” shows the ascription of the individual proteins to these pathways and the column “Highlighted in Fig” shows the highlighted proteins in the corresponding Figure. For further details see sheet “READ ME”.

**S4 Data. Lipidomic/metabolomic data of gastrocnemius muscle.**

Sheet **“GASTRO_AU”** contains list of all identified lipids and other metabolites in the tissue extracts, i.e. 506 anotated analytes. Individual mice are ordered in columns and annotated using experimental group (AJ-WA, AJ-CA, B6-WA, B6-CA) and unique sample code LM025-LM051. These data are used for multivariate analyses of lipidome of the tissue S7 Fig. Sheet **“GASTRO_pmol”** contains list of those lipids and metabolites which concentration was absolutely quantified using internal standards (see also S4 Table for the means ± SEM of the acylcarnitines data. Sheet**“GASTRO_ANOVA”** contains analytes which were significantly different among the groups according to analysis of variance (one-way ANOVA; i.e. 176 analytes). Both *p*-value (“p_raw” column) and *p*-value adjusted to false discovery rate (“p_FDR” column) are shown. Tukey’s HSD (honest significant difference) post hoc test was used to identify the individual pairs of significantly different groups. The analytes are ordered according to the *p*-value. Levels of these analytes were plotted in heat map in S7 Fig., where the order of rows corresponds to the order of rows in this Supplementary Data set. For further details see sheet “READ ME”.

***Supplementary Tables***

**S1 Table** Body weight and weight and protein of adipose tissue depots

Data from AJ and B6 mice acclimated to a thermoneutral temperature (30 °C; WA) or to cold (6 °C; CA). *significantly different in comparison to the respective thermoneutral group; ^#^ significantly different in comparison to the respective AJ group (Sidak’s multiple comparison test); s, t, and i in the last column indicate the effect of strain or temperature to which mice was adapted, or their interaction, respectively.

|  | **AJ-WA** | | | **AJ-CA** | | | **B6-WA** | | | **B6-CA** | | | **2-way** |
| --- | --- | --- | --- | --- | --- | --- | --- | --- | --- | --- | --- | --- | --- |
|  |  |  |  |  |  |  |  |  |  |  |  |  | **ANOVA** |
| **Body weight end (g)** | | | | | | | | | | | | | |
|  | 25.26 | ± | 0.4 | 22.95 | ± | 0.49 | 27.08 | ± | 0.72^#^ | 28.19 | ± | 0.62^#^ | s, i |
| **Weight of adipose tissue (mg)** | | | | | | | | | | | | | |
| **iBAT** | 67 | ± | 5 | 66 | ± | 3 | 101 | ± | 19 | 82 | ± | 6 |  |
| **rpWAT** | 120 | ± | 7 | 60 | ± | 9* | 69 | ± | 8^#^ | 59 | ± | 5 | s, t, i |
| **iWAT** | 354 | ± | 22 | 223 | ± | 22* | 230 | ± | 18^#^ | 202 | ± | 12 | s, t, i |
| **eWAT** | 328 | ± | 15 | 167 | ± | 11* | 323 | ± | 24 | 227 | ± | 15*^#^ | s, t, i |
| **Total protein per depot (mg)** | | | | | | | | | | | | | |
| **iBAT** | 0.709 | ± | 0.092 | 3.215 | ± | 0.340* | 0.209 | ± | 0.054 | 3.116 | ± | 0.236* | t |
| **rpWAT** | 1.573 | ± | 0.110 | 1.761 | ± | 0.287 | 1.222 | ± | 0.190 | 1.455 | ± | 0.136 |  |
| **iWAT** | 8.363 | ± | 0.560 | 8.677 | ± | 0.789 | 6.572 | ± | 0.507^#^ | 8.967 | ± | 0.547* | t |

**S2 Table.** Metabolic parameters and physical activity underlying cold endurance of the mice

|  |  | **AJ-WA** | | | **AJ-CA** | | | **B6-WA** | | | **B6-CA** | | | **2-way ANOVA** |
| --- | --- | --- | --- | --- | --- | --- | --- | --- | --- | --- | --- | --- | --- | --- |
| **T_b_ (°C)** | |  |  |  |  |  |  |  |  |  |  |  |  |  |
|  | **at 33 °C** | 36.17 | ± | 0.11 | 35.97 | ± | 0.16 | 36.52 | ± | 0.15 | 36.40 | ± | 0.19 | s |
|  | **at 5 °C** | 33.40 | ± | 0.40 | 34.89 | ± | 0.38 | 32.71 | ± | 0.63 | 35.50 | ± | 0.41** | t |
|  |  |  |  |  |  |  |  |  |  |  |  |  |  |  |
| **Oxygen consumption (ml/min)** | | |  |  |  |  |  |  |  |  |  |  |  |  |
|  | **at 33 °C** | 0.49 | ± | 0.06 | 0.57 | ± | 0.04 | 0.66 | ± | 0.05 | 0.74 | ± | 0.06 | s |
|  | **at 5 °C** | 2.38 | ± | 0.12 | 2.59 | ± | 0.11 | 2.52 | ± | 0.18 | 3.04 | ± | 0.10* | s, t |
|  |  |  |  |  |  |  |  |  |  |  |  |  |  |  |
| **RQ** |  |  |  |  |  |  |  |  |  |  |  |  |  |  |
|  | **at 33 °C** | 0.88 | ± | 0.04 | 0.87 | ± | 0.09 | 0.87 | ± | 0.06 | 0.83 | ± | 0.05 |  |
|  | **at 5 °C** | 0.77 | ± | 0.02 | 0.79 | ± | 0.01 | 0.78 | ± | 0.01 | 0.80 | ± | 0.01 |  |
|  |  |  |  |  |  |  |  |  |  |  |  |  |  |  |
| **Physical activity (A.U.)** | | |  |  |  |  |  |  |  |  |  |  |  |  |
|  | **at 33 °C** | 10.65 | ± | 3.85 | 2.46 | ± | 0.68 | 34.81 | ± | 12.97 | 21.63 | ± | 4.02 | s |
|  | **at 5 °C** | 16.45 | ± | 4.34 | 18.57 | ± | 2.03 | 13.19 | ± | 2.61 | 49.98 | ± | 5.68*** | s, t, i |

_____________________________________________________________________________________

Data were collected during 60 min of the measurements at (i) 33 °C; and (ii) 5 °C, starting 10 min after the animal were placed in the cold (Fig 1 and Fig. S1). Different groups of mice were used for the measurements at (i) 33 °C (*n* = 8 - 10); and (ii) 5 °C (*n* = 6 -10: AJ-WA, 10; AJ-CA, 7; B6-WA, 9; and B6-CA, 6), respectively. Data are means ± SEM. **p* < 0.05; ***p* < 0.01; and ****p* < 0.001 vs. the respective WA group (Sidak’s multiple comparison test). s, t, and i in the last column indicate the effect of strain or the acclimation temperature, or their interaction, respectively (2-way ANOVA).

**S3 Table.** Levels of acylcarnitines in skeletal muscle

Data from AJ and B6 mice acclimated to a thermoneutral temperature (30 °C; WA) or to cold (6 °C; CA). Concentrations of the analytes were evaluated in muscle extracts and and shown in pmol/mg tissue (for the source data, see S4 Data). Summary data are replotted in Fig. 8B. *significantly different in comparison to the respective thermoneutral group; ^#^ significantly different in comparison to the respective AJ group (Student t-test)

|  | **AJ - WA** | | |  | **AJ – CA** | | |  | **B6 - WA** | | |  | **B6 - CA** | | |
| --- | --- | --- | --- | --- | --- | --- | --- | --- | --- | --- | --- | --- | --- | --- | --- |
| ACar 2:0 | 11,743,711 | ± | 780,400 |  | 12,849,830 | ± | 623,611 |  | 9,357,652 | ± | 706,270^#^ |  | 8,946,918 | ± | 560,188^#^ |
| ACar 3:0-DC | 32,666 | ± | 3,672 |  | 26,043 | ± | 3,189 |  | 17,223 | ± | 1,528^#^ |  | 16,864 | ± | 1,394^#^ |
| ACar 3:0 | 94,156 | ± | 10,989 |  | 144,493 | ± | 12,192* |  | 125,230 | ± | 21,129 |  | 136,901 | ± | 14,083 |
| ACar 4:0-OH | 262,551 | ± | 15,341 |  | 361,529 | ± | 33,931* |  | 92,678 | ± | 8,062^#^ |  | 152,299 | ± | 18,026*^#^ |
| ACar 4:0 | 401,040 | ± | 46,988 |  | 697,445 | ± | 77,797* |  | 308,523 | ± | 41,918 |  | 523,384 | ± | 62,419 |
| ACar 2-M-4:0-OH | 120,094 | ± | 7,074 |  | 280,528 | ± | 15,628* |  | 103,146 | ± | 5,633 |  | 221,623 | ± | 8,489*^#^ |
| ACar 5:0 | 52,038 | ± | 11,140 |  | 158,916 | ± | 26,746* |  | 92,627 | ± | 11,963^#^ |  | 208,761 | ± | 34,938* |
| ACar 5:1 | 8,704 | ± | 1,291 |  | 30,569 | ± | 3,570* |  | 20,594 | ± | 2,664^#^ |  | 57,552 | ± | 4,612*^#^ |
| ACar 6:0 | 79,699 | ± | 11,475 |  | 108,787 | ± | 14,838 |  | 50,613 | ± | 5,885^#^ |  | 68,408 | ± | 10,339^#^ |
| ACar 8:0 | 37,215 | ± | 5,421 |  | 47,922 | ± | 5,722 |  | 28,379 | ± | 3,105 |  | 37,993 | ± | 5,033 |
| ACar 8:1 | 2,321 | ± | 236 |  | 3,478 | ± | 515* |  | 1,497 | ± | 327 |  | 2,164 | ± | 305 |
| ACar 9:0 | 471 | ± | 91 |  | 1,112 | ± | 236* |  | 3,814 | ± | 1,927 |  | 1,016 | ± | 369 |
| ACar 10:0 | 26,454 | ± | 078 |  | 33,654 | ± | 4,531 |  | 23,468 | ± | 2,360 |  | 29,378 | ± | 4,489 |
| ACar 10:1 | 9,315 | ± | 889 |  | 11,075 | ± | 1,071 |  | 6,374 | ± | 924 |  | 8,646 | ± | 1,182 |
| ACar 12:0 | 36,004 | ± | 5,658 |  | 52,978 | ± | 8,958 |  | 26,992 | ± | 2,919 |  | 42,495 | ± | 7,795 |
| ACar 12:1 | 12,383 | ± | 1,729 |  | 15,951 | ± | 2,466 |  | 11,135 | ± | 1,238 |  | 13,535 | ± | 1,931 |
| ACar 13:0 | 2,148 | ± | 389 |  | 2,977 | ± | 926 |  | 1,269 | ± | 200 |  | 1,511 | ± | 227 |
| ACar 14:0 | 178,727 | ± | 33,502 |  | 291,847 | ± | 63,495 |  | 112,553 | ± | 15,798 |  | 230,089 | ± | 47,058 |
| ACar 14:1 | 99,298 | ± | 16,582 |  | 124,100 | ± | 30,571 |  | 71,316 | ± | 10,051 |  | 91,409 | ± | 14,909 |
| ACar 14:2 | 32,454 | ± | 5,405 |  | 54,023 | ± | 12,223 |  | 27,253 | ± | 4,256 |  | 40,385 | ± | 5,564 |
| ACar 15:0 | 5,084 | ± | 938 |  | 7,317 | ± | 1,182 |  | 5,112 | ± | 897 |  | 6,356 | ± | 1,359 |
| ACar 15:2 | 10,454 | ± | 2,745 |  | 2,842 | ± | 1,467 |  | 351 | ± | 141^#^ |  | 627 | ± | 362 |
| ACar 16:0 | 652,769 | ± | 137,908 |  | 1,079,920 | ± | 240,677 |  | 488,477 | ± | 82,745 |  | 1,000,604 | ± | 242,985 |
| ACar 16:0-OH | 16,966 | ± | 2,958 |  | 24,082 | ± | 3,019 |  | 10,753 | ± | 1,539 |  | 24,474 | ± | 6,068 |
| ACar 16:1 (1) | 220,346 | ± | 43,845 |  | 300,640 | ± | 77,866 |  | 173,306 | ± | 22,340 |  | 237,712 | ± | 54,454 |
| ACar 16:1 (2) | 5,457 | ± | 884 |  | 7,481 | ± | 1,022 |  | 4,214 | ± | 486 |  | 7,784 | ± | 1,745 |
| ACar 16:2 | 55,019 | ± | 11,693 |  | 110,411 | ± | 23,737* |  | 49,311 | ± | 7,812 |  | 88,474 | ± | 17,950 |
| ACar 17:0 | 4,391 | ± | 943 |  | 6,788 | ± | 1,097 |  | 3,628 | ± | 565 |  | 5,529 | ± | 1,038 |
| ACar 17:1 | 3,759 | ± | 710 |  | 4,279 | ± | 727 |  | 4,480 | ± | 774 |  | 4,055 | ± | 800 |
| ACar 18:0 | 141,702 | ± | 34,941 |  | 394,093 | ± | 80,724* |  | 83,121 | ± | 11,791 |  | 285,582 | ± | 59,166* |
| ACar 18:1 (1) | 791,646 | ± | 177,150 |  | 1,096,141 | ± | 252,270 |  | 703,580 | ± | 128,993 |  | 831,333 | ± | 181,287 |
| ACar 18:1 (2) | 3,156 | ± | 445 |  | 6,492 | ± | 719* |  | 2,253 | ± | 264 |  | 4,937 | ± | 952* |
| ACar 18:1-OH | 24,092 | ± | 3,571 |  | 33,615 | ± | 5,624 |  | 19,429 | ± | 2,857 |  | 27,343 | ± | 6,296 |
| ACar 18:2 (1) | 309,347 | ± | 67,950 |  | 624,554 | ± | 138,349* |  | 315,720 | ± | 64,362 |  | 486,571 | ± | 107,561 |
| ACar 18:2 (2) | 13,224 | ± | 1,655 |  | 16,980 | ± | 2,637 |  | 12,796 | ± | 1,721 |  | 16,558 | ± | 3,600 |
| ACar 18:3 (1) | 14,007 | ± | 2,844 |  | 29,046 | ± | 5,950* |  | 15,184 | ± | 2,068 |  | 28,310 | ± | 4,858 |
| ACar 18:3 (2) | 3,347 | ± | 547 |  | 6,136 | ± | 1,067* |  | 3,311 | ± | 560 |  | 5,964 | ± | 1,332 |
| ACar 19:0 | 1,457 | ± | 339 |  | 2,678 | ± | 513* |  | 1,027 | ± | 164 |  | 2,242 | ± | 534* |
| ACar 19:1 | 1,852 | ± | 423 |  | 2,497 | ± | 506 |  | 1,731 | ± | 345 |  | 1,812 | ± | 396 |
| ACar 20:0 | 6,440 | ± | 1,942 |  | 40,902 | ± | 10,331* |  | 3,688 | ± | 411 |  | 23,513 | ± | 5,966* |
| ACar 20:1 | 46,013 | ± | 13,775 |  | 82,834 | ± | 22,494 |  | 28,743 | ± | 4,264 |  | 55,789 | ± | 16,941 |
| ACar 20:2 | 24,420 | ± | 7,099 |  | 59,605 | ± | 14,013* |  | 16,969 | ± | 2,275 |  | 41,759 | ± | 13,822 |
| ACar 20:3 (1) | 10,572 | ± | 2,065 |  | 26,469 | ± | 5,273* |  | 10,534 | ± | 2,387 |  | 16,821 | ± | 3,913 |
| ACar 20:3 (2) | 3,138 | ± | 681 |  | 4,784 | ± | 942 |  | 2,493 | ± | 575 |  | 2,198 | ± | 472^#^ |
| ACar 20:4 | 29,438 | ± | 6,486 |  | 81,420 | ± | 19,207* |  | 24,944 | ± | 6,394 |  | 37,852 | ± | 8,073^#^ |
| ACar 20:5 | 1,488 | ± | 304 |  | 6,488 | ± | 1,839* |  | 1,847 | ± | 455 |  | 4,441 | ± | 1,087* |
| ACar 22:2 | 1,306 | ± | 568 |  | 3,142 | ± | 579* |  | 708 | ± | 81 |  | 1,848 | ± | 587 |
| ACar 22:4 | 4,816 | ± | 1,236 |  | 17,480 | ± | 4,802* |  | 3,136 | ± | 872 |  | 6,221 | ± | 1,896^#^ |
| ACar 22:5 | 972 | ± | 247 |  | 5,090 | ± | 1,681* |  | 1,509 | ± | 491 |  | 3,188 | ± | 979 |
| ACar 22:6 | 2,558 | ± | 745 |  | 5,863 | ± | 1,812 |  | 4,117 | ± | 1,285 |  | 4,408 | ± | 913 |

**S4 Table.** List of primer sequences

| **Gene name** | **Gene ID** | **Forward primer** | **Reverse primer** |
| --- | --- | --- | --- |
| *Actb* | 11461 | GAACCCTAAGGCCAACCGTGAAAAGAT | ACCGCTCGTTGCCAATAGTGATG |
| *Eef2* | 13629 | GAAACGCGCAGATGTCCAAAAGTC | GCCGGGCTGCAAGTCTAAGG |
| *Hprt* | 15452 | GCTGAGGCGGCGAGGGAGAG | GCTAATCACGACGCTGGGACTGC |
| *Rn18S* | 19791 | GCCCGAGCCGCCTGGATAC | CCGGCGGGTCATGGGAATAAC |
| *Sln* | 66402 | AAGCTAAGGCTCACTGGCTGGC | TCACAATGCCTGACACACCGCT |
| *Atp2a1* | 11937 | GTGGGCGAGGTGGTCTGTATCTTC | AGTAGCCGGGAGCCCATCAGTCAC |
| *Atp2a2* | 11938 | CACATGCACGCACCCGAACA | CGTGGAACCTTTGCCGCTCATT |
| *Ucp1* | 22227 | CACGGGGACCTACAATGCTTACAG | GGCCGTCGGTCCTTCCTT |

Abbreviations: *Actb*, actin beta; *Eef2,* eukaryotic translation elongation factor 2; *Hpr*t, hypoxanthine guanine phosphoribosyl transferase; *Rn18S*, 18S ribosomal RNA; *Sln*, sarcolipin; *Atp2a1*, ATPase, Ca++ transporting, cardiac muscle, fast twitch 1 (SERCA1); *Atp2a2*, ATPase, Ca++ transporting, cardiac muscle, slow twitch 2 (SERCA2); *Ucp1*, uncoupling protein 1.

***Supplementary Figures***

**S1 Fig. Lower cold endurance of B6 compared with AJ warm-acclimated mice and rescue of the endurance by CA – mean curves**

Whole-body measurements were performed using AJ and B6 mice acclimated to a thermoneutral housing temperature (30 °C; WA) or to cold (6 °C; CA). Measurements were performed at 33 °C and 5 °C, respectively, using different groups of mice. See S2 Table for further details. **(A** and **B)** T_b_ of WA (**A**) and CA mice (**B**) measured at 33 °C (left part of the graphs) or when exposed to 5 °C (right part of the graphs); for the individual mice data, see Fig 1A and 1B.

**(C** and **D)** Oxygen consumption of WA (**C**) and CA mice (**D**) measured at 33 °C (left part of the graphs) or when exposed to 5 °C (right part of the graphs); for the individual mice data, see Fig 1C and 1D.

Arrows, the time when the individual mice exhibiting hypothermy were rescued from cold (see Fig 1).

**
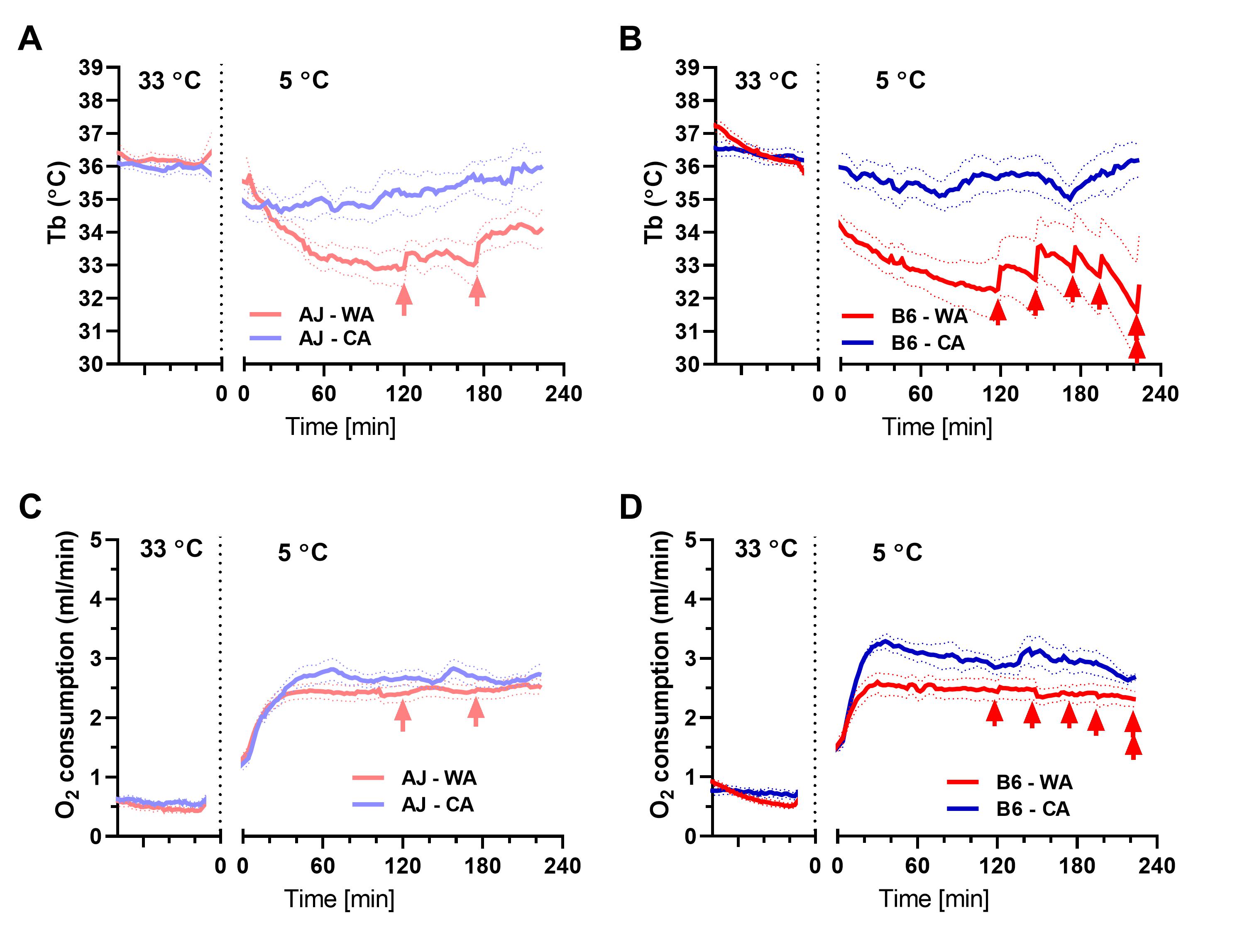
**

**S2 Fig. Thermal physiology of AJ and B6 mice during CA**

AJ and B6 mice at thermoneutrality and during subsequent acclimation to cold (after 2 days and 7 days at 8 °C).

(**A**) Representative infrared pictures of AJ and B6 at 30 °C and during acclimation to 8 °C. (**B**) Quantification of the hair surface temperature in the upper back area (see panel **A**). (**C**) Thermal conductance of AJ and B6 mice adapted (for 2 weeks) and measured at ambient temperature of 8, 22, and 30°C.

No difference between strains according to 2-way ANOVA. (**B, C**) Values for each data point and number of animals in each group can be found in S1 Data.

**S3 Fig. Effects of acclimation temperature and mouse strain on the function of UCP1 in iBAT mitochondria**

Oxygen consumption of isolated iBAT mitochondria (reference below) was measured using Oroboros Oxygraph (Austria) at 30 °C, with 0.2 mg mitochondrial suspension protein in 2 ml of K-medium (see Materials and Methods) containing 10 μM cytochrome c, 10 mM pyruvate and 2.5 mM malate. Several additions of GDP (to increase its concentration stepwise by 0.5 mM) were made, to inhibit UCP1 activity

**(A)** A representative measuring curve demonstrating induction of maximal respiratory rate using pyruvate and malate (pyr +mal) and inhibition of UCP1 protonophoric activity using GDP.

**(B)** Mean values of respiratory rate in the presence of pyruvate and malate (the maximal rate), and under inhibition of UCP1 activity by GDP (data corresponding to representative curves in **A**).

*n* = 4 in AJ-WA, 6 in AJ-CA, 5 in B6-WA, and 10 in B6-CA. * difference vs. the respective CA group, # difference vs. the AJ mice at the respective temperature (two-way ANOVA and Tukey's multiple comparison test). (**B**) Values for each data point can be found in S1 Data.

**Reference:** Shabalina IG, Vrbacky M, Pecinova A, Kalinovich AV, Drahota Z, Houstek J, et al. ROS production in brown adipose tissue mitochondria: the question of UCP1-dependence. *Biochim Biophys Acta.* 2014;1837(12):2017-30.

**
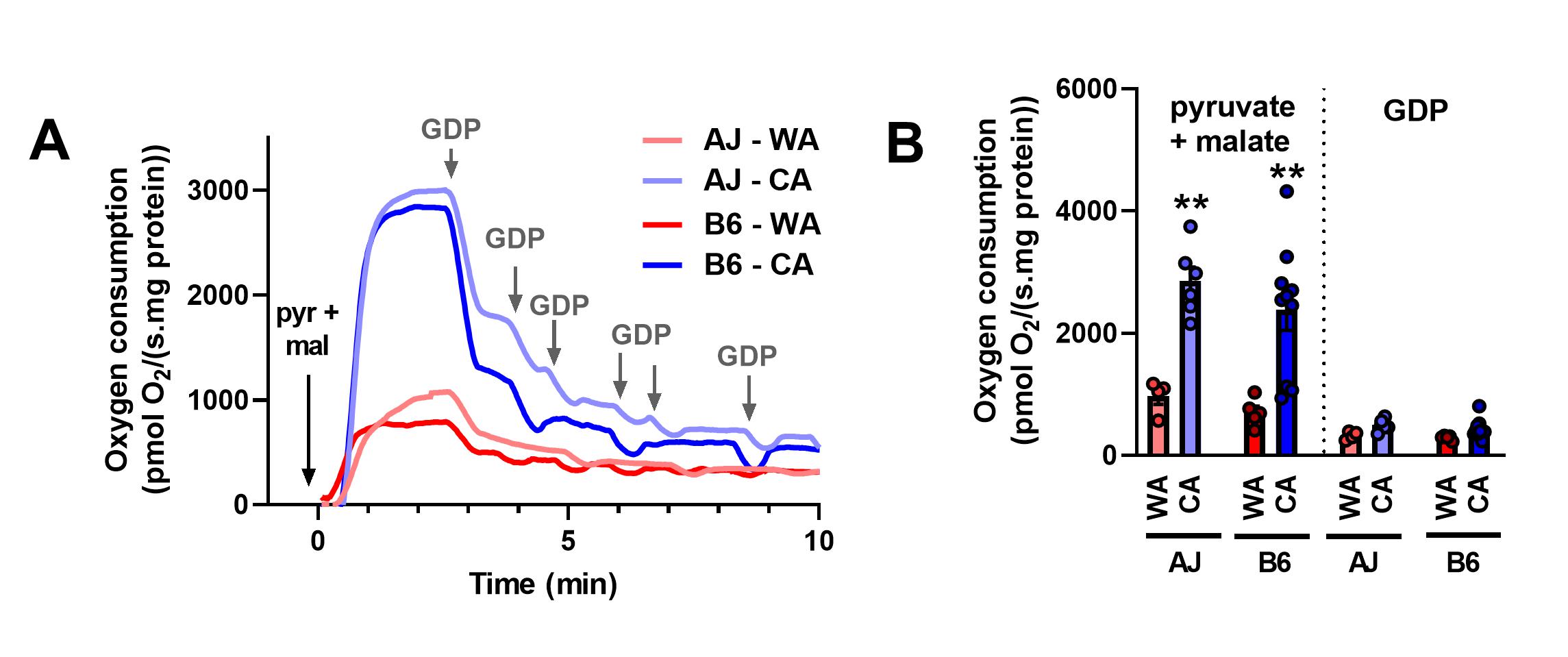
**

**S4 Fig. Effect of the mouse strain on iBAT proteome**

Analyses of iBAT proteome of AJ and B6 mice acclimated to a thermoneutral temperature (30°C; WA) or to cold (6 °C; CA) was performed using mass-spectrometry label-free quantification (MS-LFQ; *n* = 4).

**(A** and **B)** Volcano plots to demonstrate the difference in quantitative proteome composition between the AJ and the B6 mice, based on all 3501 proteins detected; S2 and S3 Data); plotted separately for the WA and the CA mice (upper and lower panels, respectively). Significantly regulated proteins (i.e. *p*-value < 0.05; fold change >1.5) were (i) indicated by black dots (in AJ-WA vs. B6-WA comparison: 27 proteins upregulated in AJ and 20 proteins upregulated in B6; in AJ-CA vs. B6-CA comparison: 17 proteins upregulated in AJ and 35 proteins upregulated in B6; see S3 Data); (ii) ascribed to major metabolic pathways using KEGG database (see S3 Data); and (iii) color-coded according their involvement in metabolism. For the protein codes, see the entry name of UniProt database (used here without the name of the organism (e.g. UCP1 is originally UCP1_MOUSE; see S3 Database).

**
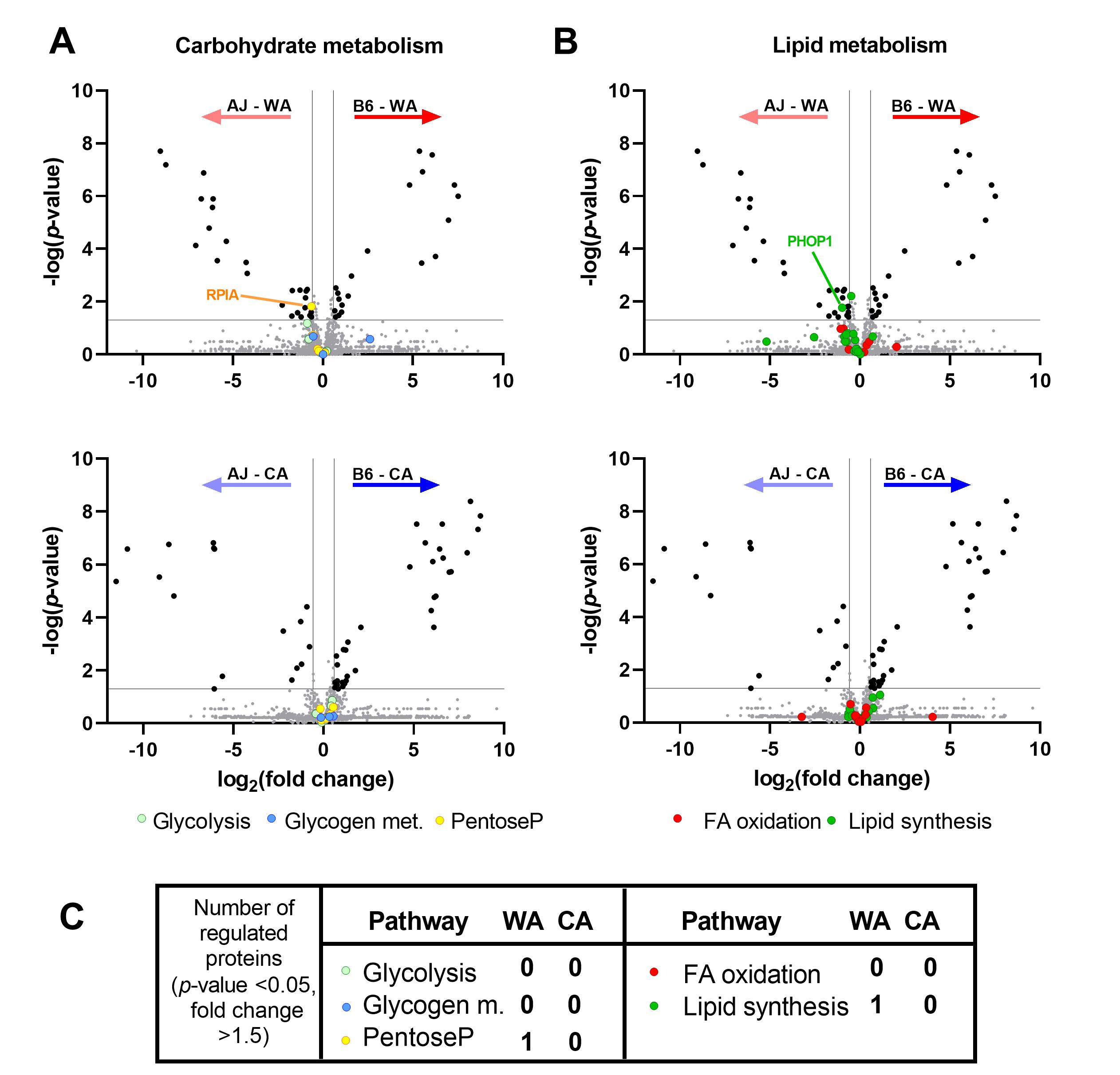
**(**C**) Number of differentially regulated proteins/enzymes engaged in selected metabolic pathways in mice of both strains (see S3 Data). For the effect of the acclimation temperature, see Fig 5 D-F.

**S5 Fig. Effects of acclimation temperature and mouse strain on OXPHOS proteins in iBAT**

AJ and B6 mice acclimated to a thermoneutral temperature (30 °C; WA) or to cold (6 °C; CA) were used for analysis of iBAT proteome using mass-spectrometry label-free quantification (MS-LFQ; *n* = 4).

Volcano plots to demonstrate the difference in quantitative proteome composition between AJ-WA and AJ-CA, B6-WA and B6-CA, AJ-WA and B6-WA, and AJ-CA and B6-CA, respectively. UCP1 and all significantly regulated OXPHOS proteins (*p*-value<0.05) are labelled with the entry name from UNIPROT database (as in Fig 5D and E). For details about all significantly regulated proteins (labelled by black dots), see S3 Data and Figs. 5D-E.

**
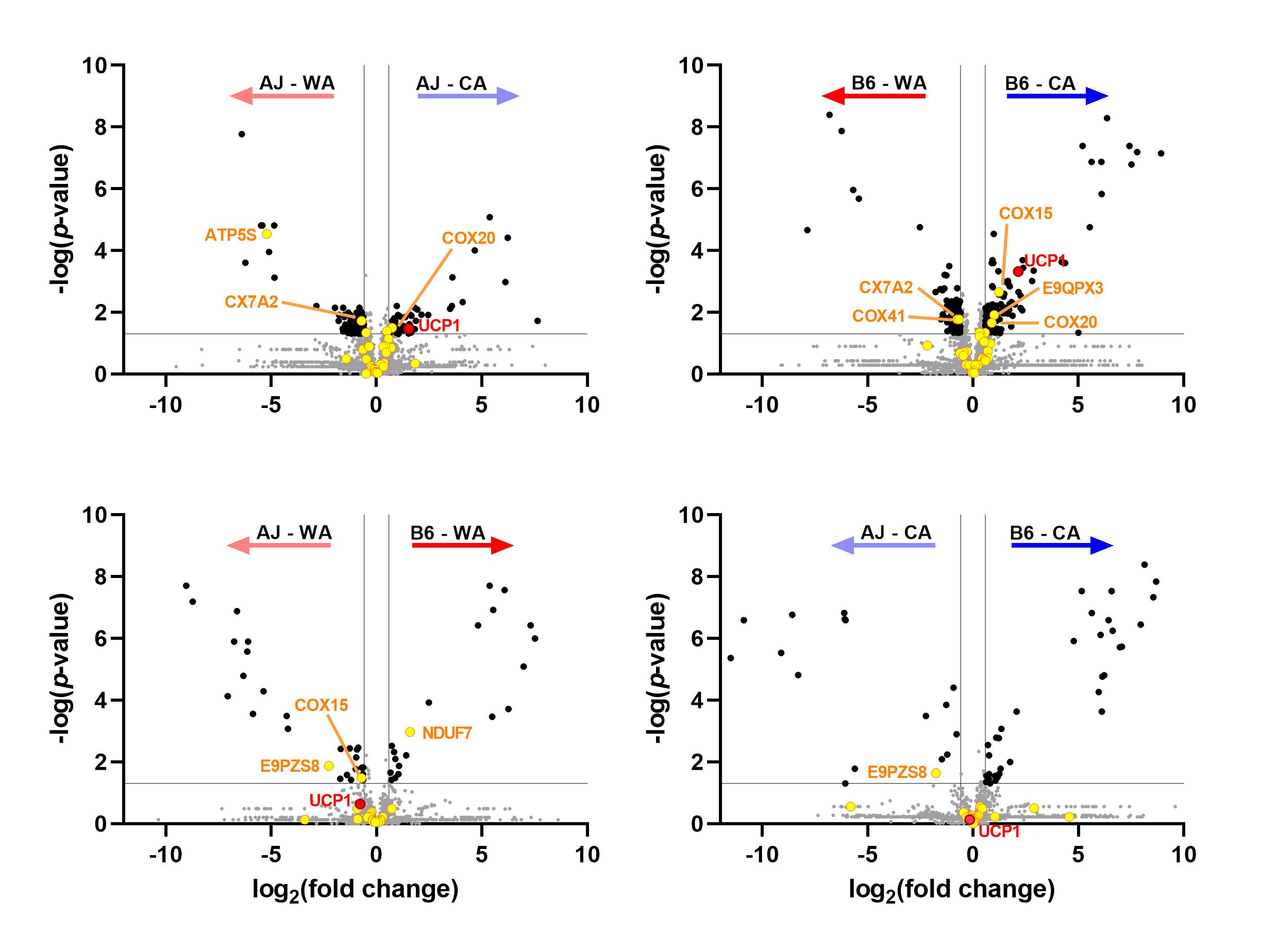
**

**S6 Fig.** Levels of enzymes and protein subunits involved in DNL and oxidative metabolism of lipids in muscle (A.U.; proteomic data)

**(A)** Proteins involved in DNL. **(B)** Proteins involved in FAox. **(C)** Subunits of OXPHOS complexes affected by CA in AJ mice (based on t-test as shown in volcano plot in Fig. 6B). **(D)** Subunits of cytochrome oxidase complex.

*n* = 4. Significant effect of strain is labelled by s, significant effect of temperature is labelled by t, and significant interaction between the strain and temperature effects is highlighted (2-way ANOVA). **(A, B, C, D)** Values for each data point are included in S2 Data.

**
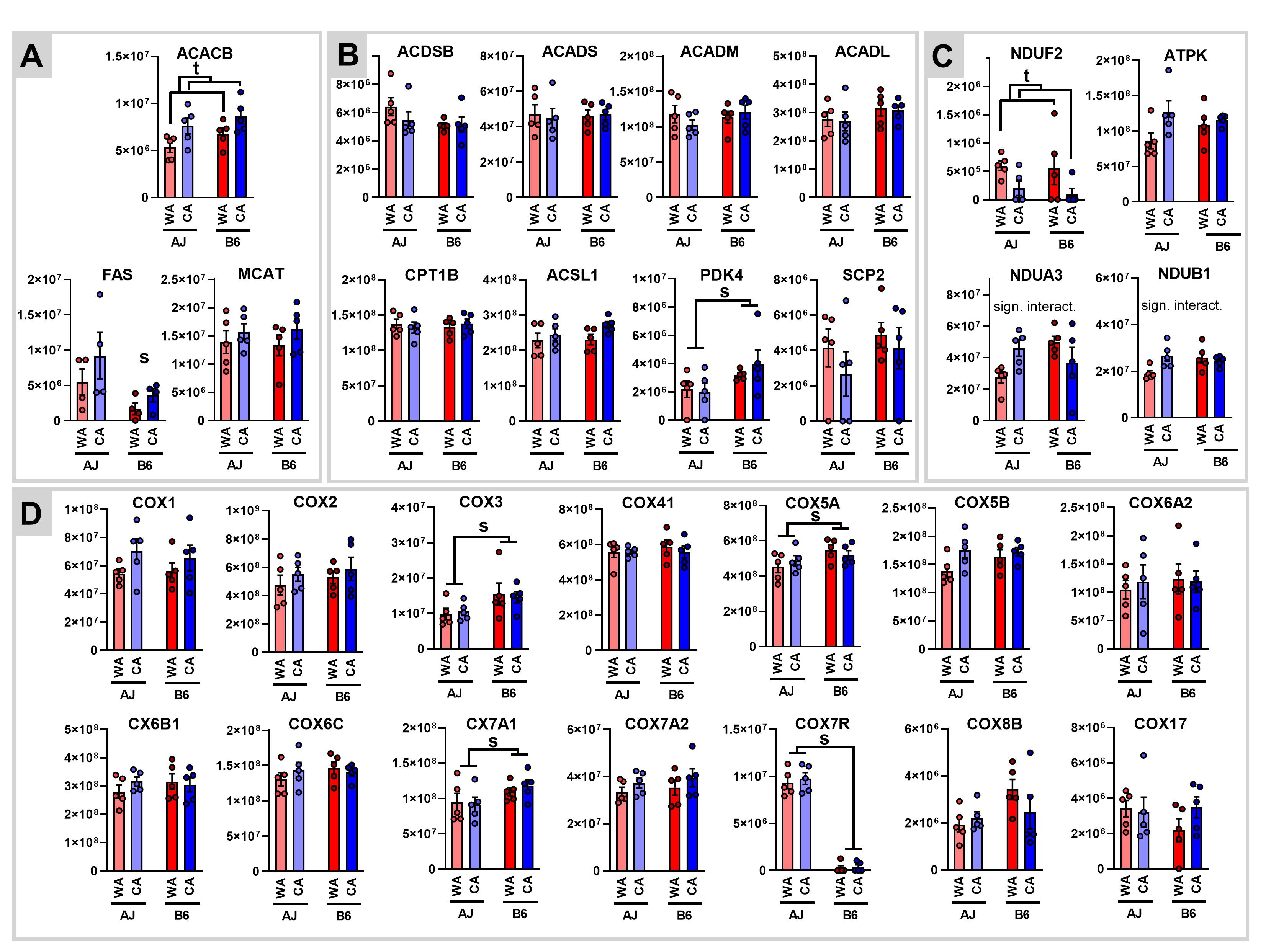
**

**S7 Fig. Whole lipidome in skeletal muscle**

AJ and B6 mice exposed to thermoneutral housing temperature (30°C) or to cold (6 °C) for 7 days (*n* = 5-6). Hierarchial clustering of analytes in muscle extracts. Each column represents an individual animal (experimental group is indicated by color code above the column), each row represents an individual analyte ordered according to analyte classes. Only analytes differing significantly among the experimental groups (one-way ANOVA) were considered (i.e. 176 out of 506 annotated analytes detected; S4 Data). Mice were automatically clustered using MetaboAnalyst (v 4.0 and 5.0) software (reference below) as indicated by dendrograms above and left of the plot. Hue represents the autoscaled t-test/ANOVA score. AC – acylcarnitines; Cer – ceramides; CL – cardiolipins; Non-polar – mono-, di-, and triacylglycerols; PL – phospholipids; SM – sphingomyelins.

**
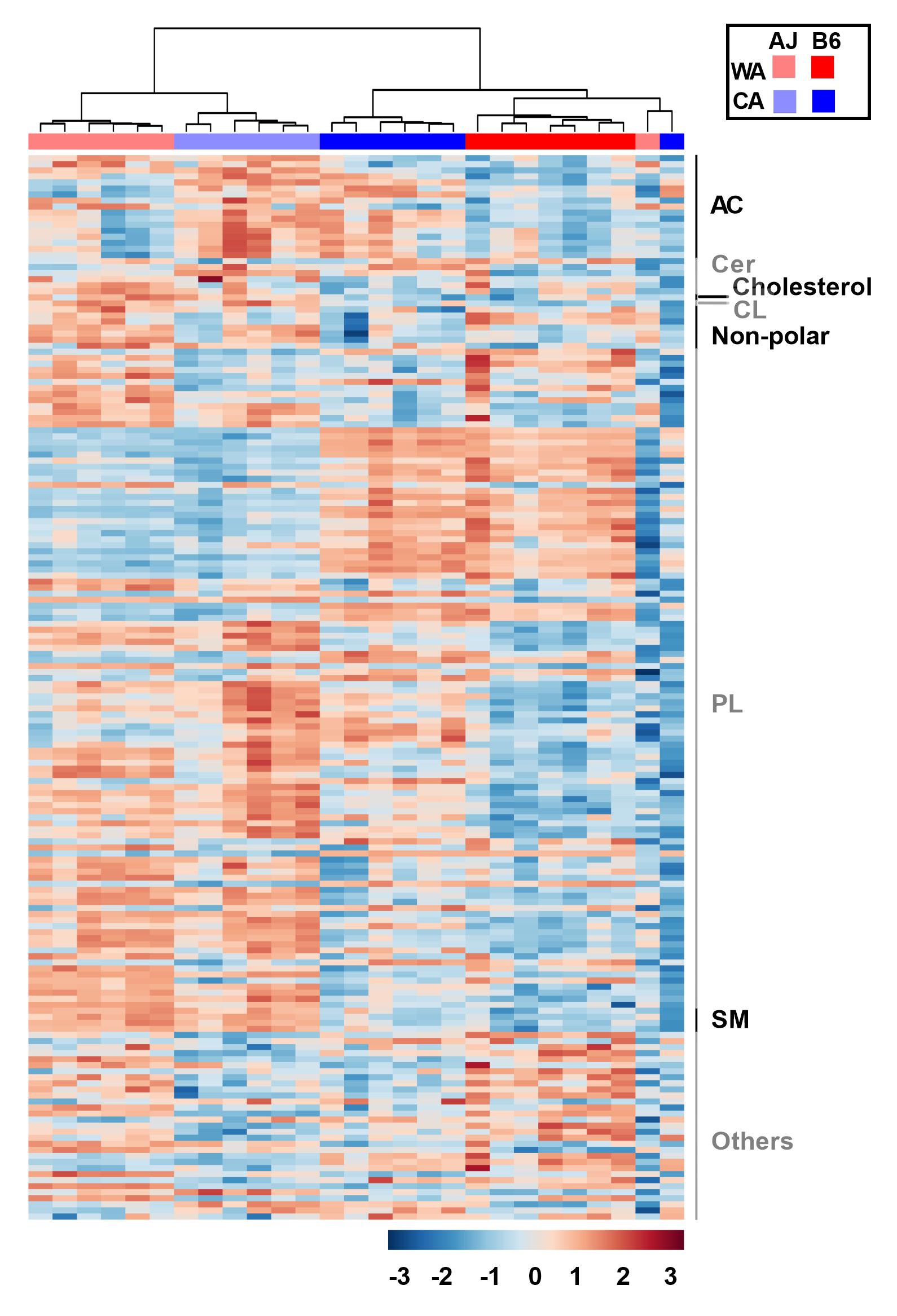
Reference:** Chong J, Soufan O, Li C, Caraus I, Li S, Bourque G, et al. MetaboAnalyst 4.0: towards more transparent and integrative metabolomics analysis. Nucleic Acids Res. 2018;46(W1):W486-W94.

**S8 Fig. Measurement of palmitoyl carnitine oxidation**

A representative respiratory curves of palmitoyl carnitine (PC) oxidation of muscle homogenates. For details, see Methods. The black bar around Time = 5 min, measurement of the peak values of oxygen consumption, shown in Fig. 8D.

**
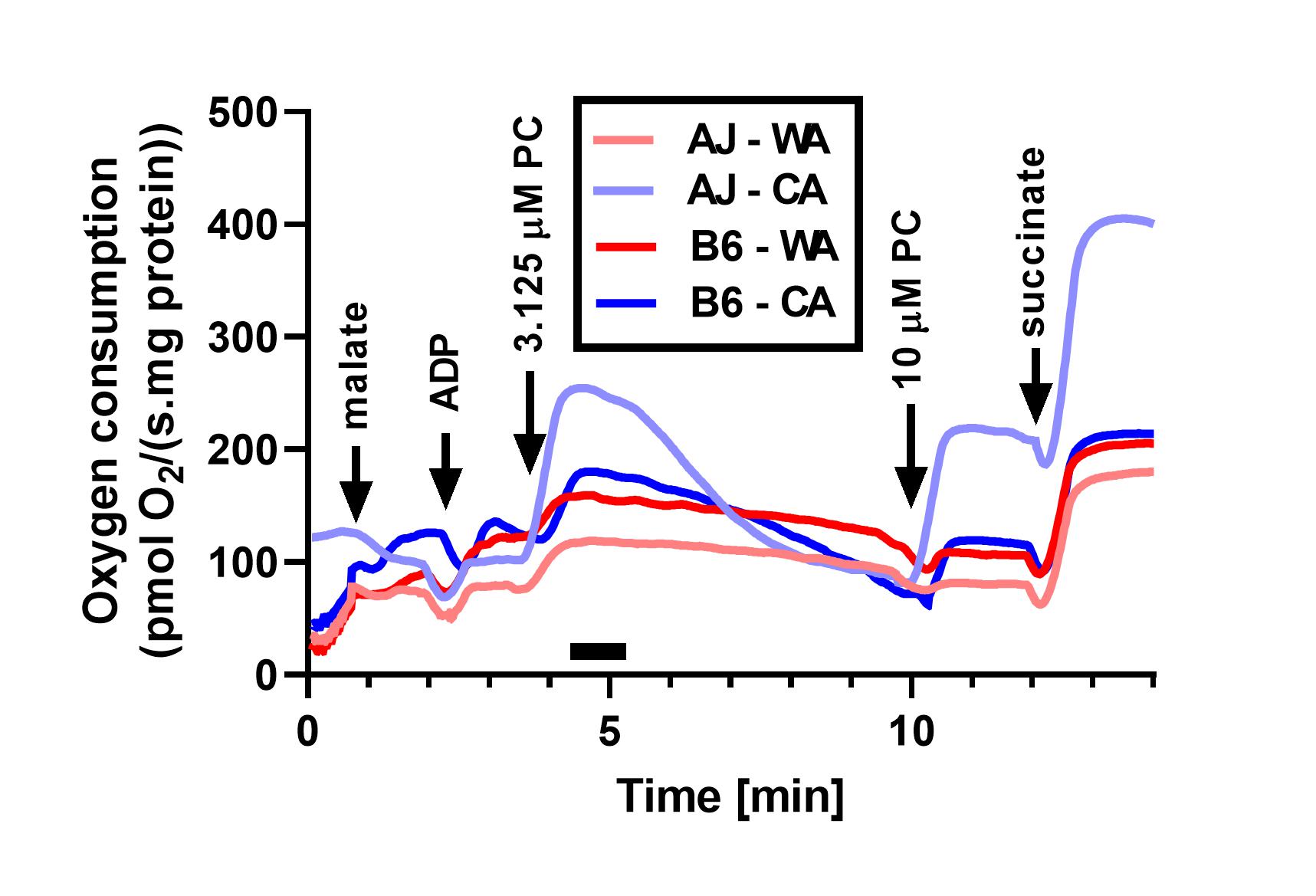
**
